# Mycorrhizal strategy of non-native plants varies with biome and disturbance

**DOI:** 10.1038/s41559-026-03144-9

**Published:** 2026-08-17

**Authors:** Sara Giulia Cazzaniga, Thomas Lauber, Johan van den Hoogen, Evelyn M. Beaury, C. Guillermo Bueno, James D. Bever, Gabriella Damasceno, Jane Catford, Jonathan Lenoir, Adam Martin, Akira S. Mori, Alexander Novakovskiy, Alvaro G. Gutiérrez, Ana González-Robles, André Luís de Gasper, Angela Moles, Angela Stanisci, Anikó Csecserits, Anke Jentsch, Anna Kuzemko, Antonio J. Perea, Ashish Nerlekar, Avishkar Munje, Behlül Güler, Borja Jiménez-Alfaro, Bruno Hérault, Bruno X. Pinho, Chris Baraloto, Christian Rossi, Daniel Hending, Daniel C. Laughlin, Dave Rogers, David Schellenberger Costa, Dinesh Thakur, Domas Uogintas, Eduardo Chacón-Madrigal, Esteban Alvarez-Davila, Evan Weiher, Evert Thomas, Fernando Gonçalves, Flávio Rodrigues, Francesco Maria Sabatini, Frank M. Schurr, Franz Essl, Georg Zizka, Gianmaria Bonari, Grzegorz Swacha, Han Y. H. Chen, Helge Bruelheide, Henry Ford, Hua-Feng Wang, Idoia Biurrun, Isabelle Aubin, Iwona Dembicz, Jacob Willie, Jan Altman, Jens-Christian Svenning, Jeremy W. Lichstein, Jesper Erenskjold Moeslund, Jiri Dolezal, Johannes H. C. Cornelissen, John Hunter, Julie Messier, Jürgen Dengler, Jürgen Homeier, Kate H. Orwin, Kirill Korznikov, Koenraad Van Meerbeek, Manuel J. Macía, Marco Schmidt, Marco Varricchione, Marcos Bergmann Carlucci, Marko Spasojevic, Melisa A. Giorgis, Milan Chytrý, Mohamed A. El-Sheikh, Mohamed Z. Hatim, Nathan J. B. Kraft, Neha Mohanbabu, Oliver L. Phillips, Peter B. Reich, Remigiusz Pielech, Ricard Arasa-Gisbert, Riccardo Guarino, Riccardo Testolin, Rubén Tarifa, Sharif A. Mukul, Shyam S. Phartyal, Sylvain Schmitt, Sylvia Haider, Tetiana Dziuba, Tomas Domingues, Udayangani Liu, Valentin Golub, Vasco Silva, Veronika Fontana, Vigdis Vandvik, W. Daniel Kissling, Zvjezdana Stančić, Marten Winter, Thomas W. Crowther, Camille S. Delavaux

**Affiliations:** 1 Environmental Systems Science, ETH Zürich, Zurich, Switzerland; 2 Center for Conservation and Restoration Ecology, New York Botanical Garden, Bronx, NY USA; 3 Pyrenean Institute of Ecology, CSIC (Spanish Research Council), Jaca, Spain; 4 Department of Ecology and Evolutionary Biology, University of Kansas, Lawrence, KS USA; 5 Institute of Biology/Geobotany and Botanical Garden, Martin Luther University, Halle, Germany; 6 German Centre for Integrative Biodiversity Research (iDiv), Leipzig, Germany; 7 Department of Geography, King’s College London, London, UK; 8 Fenner School of Environment & Society, Australian National University, Canberra, Australian Capital Territory Australia; 9 Ecologie et Dynamiques des Systèmes Anthropisés (EDYSAN), UMR CNRS 7058, University of Picardie Jules Verne, Amiens, France; 10 Department of Physical and Environmental Sciences, University of Toronto Scarborough, Toronto, Ontario Canada; 11 Research Center for Advanced Science and Technology, University of Tokyo, Tokyo, Japan; 12 Syktyvkar, Russia; 13 Department of Environmental Sciences and Renewable Natural Resources, Faculty of Agronomic Sciences, University of Chile, Santiago, Chile; 14 Department of Animal Biology, Plant Biology and Ecology, University of Jaén, Campus Lagunillas SN, Jaén, Spain; 15 Regional University of Blumenau, Blumenau, Brazil; 16 Evolution & Ecology Research Centre, School of Biological, Earth and Environmental Sciences, UNSW Sydney, Sydney, New South Wales Australia; 17 Department of Biosciences and Territory, University of Molise, Termoli, Italy; 18 HUN-REN Centre for Ecological Research, Institute of Ecology and Botany, Vácrátót, Hungary; 19 Disturbance Ecology and Vegetation Dynamics, University of Bayreuth, Bayreuth, Germany; 20 Department of Geobotany and Ecology, M.G. Kholodny Institute of Botany, National Academy of Sciences of Ukraine, Kyiv, Ukraine; 21 Plant Biology, Michigan State University, East Lansing, MI USA; 22 Savanna Science Foundation, Pune, India; 23 Symbiosis Centre for Climate Change and Sustainability (SCCCS), Symbiosis International (Deemed) University, Pune, India; 24 Biology Education, Dokuz Eylül University, Izmir, Turkey; 25 Biodiversity Research Institute (IMIB), University of Oviedo, Mieres, Spain; 26 Department of Organismal and Systems Biology, University of Oviedo, Oviedo, Spain; 27 Forests and Societies, CIRAD Baillarguet, Montpellier, France; 28 Institute of Plant Sciences, University of Bern, Bern, Switzerland; 29 Institute of Environment, Florida International University, Miami, FL USA; 30 Swiss National Park, Zernez, Switzerland; 31 Department of Biology, University of Oxford, Oxford, UK; 32 Department of Botany, University of Wyoming, Laramie, WY USA; 33 Biological Sciences Department, University of Wisconsin-Parkside, Kenosha, WI USA; 34 Systematic Botany and Functional Diversity Lab, Faculty of Life Sciences, Leipzig University, Leipzig, Germany; 35 Institute of Botany of the Czech Academy of Sciences, Průhonice, Czech Republic; 36 State Scientific Research Institution Nature Research Centre, Vilnius, Lithuania; 37 Center for Research in Biodiversity and Tropical Ecology, University of Costa Rica, San Pedro Montes de Oca, Costa Rica; 38 Ecosystem Services and Climate Change Research Group, Fundación Con Vida (Colombia), Medellín, Colombia; 39 Department of Biology, University of Wisconsin—Eau Claire, Eau Claire, WI USA; 40 Bioversity International, Lima, Peru; 41 Department of Evolutionary Biology and Environmental Studies, University of Zurich Switzerland, Zurich, Switzerland; 42 UNEMAT, Nova Xavantina, Brazil; 43 BIOME Lab, Department of Biological, Geological and Environmental Sciences (BiGeA), Alma Mater Studiorum University of Bologna, Bologna, Italy; 44 Institute of Landscape and Plant Ecology, University of Hohenheim, Stuttgart, Germany; 45 Division of BioInvasions, Macroecology & Global Change, University of Vienna, Vienna, Austria; 46 Department of Botany and Molecular Evolution, Senckenberg Frankfurt and Goethe University, Frankfurt, Germany; 47 Department of Life Sciences, University of Siena, Siena, Italy; 48 Botanical Garden, University of Wrocław, Wrocław, Poland; 49 College of Grassland Science, Inner Mongolia Agricultural University, Hohhot, China; 50 Faculty of Natural Resources Management, Lakehead University, Thunder Bay, Ontario Canada; 51 Institute for Global Change Biology, School for Environment and Sustainability, University of Michigan, Ann Arbor, MI USA; 52 School of Design, Bath Spa University, Bath, UK; 53 College of Tropical Agriculture and Forestry, Hainan University, Haikou, China; 54 Department of Plant Biology and Ecology, University of the Basque Country UPV/EHU, Bilbao, Spain; 55 Great Lakes Forestry Centre, Canadian Forest Service, Natural Resources Canada, Sault Ste Marie, Ontario Canada; 56 Institute of Environmental Biology, Faculty of Biology, University of Warsaw, Warsaw, Poland; 57 Centre for Research and Conservation, Royal Zoological Society of Antwerp, Antwerp, Belgium; 58 Institute of Botany, the Czech Academy of Sciences, Průhonice, Czech Republic; 59 Faculty of Forestry and Wood Sciences, Czech University of Life Sciences, Prague, Czech Republic; 60 Center for Ecological Dynamics in a Novel Biosphere (ECONOVO), Aarhus University, Aarhus C, Denmark; 61 Department of Biology, University of Florida, Gainesville, FL USA; 62 Department of Ecoscience, Aarhus University, Aarhus C, Denmark; 63 Faculty of Science, University of South Bohemia, České Budějovice, Czech Republic; 64 Systems Ecology, A-LIFE, Vrije University Amsterdam, Amsterdam, the Netherlands; 65 School of Environmental and Rural Science, University of New England, Armidale, New South Wales Australia; 66 Department of Biology, University of Waterloo, West Waterloo, Ontario Canada; 67 Institut für Umwelt und Natürliche Ressourcen, Zurich University of Applied Sciences (ZHAW), Wädenswil, Switzerland; 68 Bayreuth Center of Ecology and Environmental Research (BayCEER), University of Bayreuth, Bayreuth, Germany; 69 Faculty of Resource Management, HAWK University of Applied Sciences and Arts, Göttingen, Germany; 70 Bioeconomy Sciences Institute, Manaaki Whenua Group, Lincoln, New Zealand; 71 Department of Earth and Environmental Sciences, KU Leuven, Leuven, Belgium; 72 Plant Institute, KU Leuven, Leuven, Belgium; 73 Departament of Biology, Autonomous University of Madrid, Madrid, Spain; 74 Center for Research on Biodiversity and Global Change (CIBC-UAM), Autonomous University of Madrid, Madrid, Spain; 75 Palmengarten, Frankfurt, Germany; 76 Department of Biosciences and Territory, University of Molise, Isernia, Italy; 77 Departament of Botany, University of the Federation of Paraná—UFPR, Curitiba, Brazil; 78 Department of Evolution, Ecology and Organismal Biology, University of California Riverside, Riverside, CA USA; 79 Instituto Multidisciplinario de Biología Vegetal (IMBIV CONICET-UNC), Córdoba, Argentina; 80 Department of Botany and Zoology, Faculty of Science, Masaryk University, Brno, Czech Republic; 81 Botany and Microbiology Department, King Saud University, Riyadh, Saudi Arabia; 82 Plant Ecology and Nature Conservation Group, Wageningen University and Research, Wageningen, the Netherlands; 83 Department of Ecology and Evolutionary Biology, University of California Los Angeles (UCLA), Los Angeles, CA USA; 84 Copernicus institute of Sustainable Development, Utrecht University, Utrecht, the Netherlands; 85 School of Geography, University of Leeds, Leeds, UK; 86 Institute for Global Change Biology, University of Michigan, Ann Arbor, MI USA; 87 Department of Forest Resources, University of Minnesota, St Paul, MN USA; 88 Institute of Botany, Faculty of Biology, Jagiellonian University, Kraków, Poland; 89 Facultad de Estudios Superiores Iztacala, Universidad Nacional Autónoma de México, Tlanepantla de Baz, Mexico; 90 Department of Biological Chemical and Pharmaceutical Science and Technology (STEBICEF), Botanical Unit, University of Palermo, Palermo, Italy; 91 Estación Experimental de Zonas Áridas EEZA, CSIC, Almería, Spain; 92 Department of Environment and Development Studies, United International University, Dhaka, Bangladesh; 93 Tropical Forests and People Research Centre, University of the Sunshine Coast, Moroochydore, Queensland Australia; 94 Department of Forestry, Mizoram University, Aizawl, India; 95 Forêts et Sociétés, CIRAD Baillarguet, Montpellier, France; 96 Institute of Ecology, Leuphana University of Lüneburg, Lüneburg, Germany; 97 University of São Paulo, FFCLRP, Ribeirão Preto, Brazil; 98 Digital Revolution, Kew Wakehurst, Ardingly, UK; 99 Togliatti, Russia; 100 Egas Moniz Center for Interdisciplinary Research (CiiEM), Egas Moniz School of Health and Science, Caparica, Portugal; 101 Institute for Alpine Environment, Eurac Research, Bolzano, Italy; 102 CeSAM Centre for Sustainable Area Management, University of Bergen, Bergen, Norway; 103 Institute for Biodiversity and Ecosystem Dynamics (IBED), University of Amsterdam, Amsterdam, the Netherlands; 104 Faculty of Geotechnical Engineering, University of Zagreb, Varaždin, Croatia; 105 Environmental Sciences and Engineering, Biological and Environmental Science and Engineering Division, King Abdullah University of Science and Technology, Thuwal, Kingdom of Saudi Arabia; 106 BRANCH Institute, Zug, Switzerland; 107 Department of Terrestrial Ecology, Netherlands Institute of Ecology (NIOO-KNAW), Wageningen, the Netherlands

**Keywords:** Ecosystem ecology, Invasive species, Biogeography, Microbial ecology, Ecological modelling

## Abstract

Predicting which non-native plant species will become established and where is critical for conserving and managing biodiversity. Theory suggests that the mycorrhizal strategy of non-native plants may predict their establishment success. Here we combine a global dataset of 440,788 vegetation plots with data on plant native status and mycorrhizal type to assess mycorrhizal strategy of non-native plants. The mycorrhizal strategy of non-native plants varies strongly across biomes. Across grassland and desert biomes, non-native species are more frequently non-mycorrhizal than native species, whereas in other biomes non-native species are more likely to be mycorrhizal, most commonly arbuscular-mycorrhizal. Disturbance type and intensity are key predictors of mycorrhizal strategy of non-native species, as mycorrhizal species are favoured by landscape modification and non-mycorrhizal species by natural and human-caused disturbance events. Facultatively mycorrhizal species are consistently under-represented among non-native plants compared with natives, suggesting that symbiotic flexibility does not confer an advantage for non-natives as previously expected. Our study shows that non-native mycorrhizal strategy varies across biogeographical contexts and disturbance, highlighting the need for region-specific prevention and management approaches to plant species introductions.

## Abstract (web summary)

Mycorrhizal strategy could be one of the factors that explain why certain non-native species become successful invaders. This study tests this idea at the global scale and explores geographic patterns and putative drivers of mycorrhizal strategies of non-native naturalized plants.

## Main

Non-native plant species introductions pose a major threat worldwide, leading to biodiversity loss and ecosystem degradation^1–3^. Non-native plant species can outcompete native flora, disrupt trophic interactions and modify nutrient cycling, ultimately reshaping entire ecosystems^4–6^ and causing high financial costs^7,8^. A common approach to predict non-native plant invasion success is to evaluate traits linked to successful colonization, resource acquisition and fitness of plant species^9,10^. However, such approaches have shown limited predictive power as invasion outcomes are highly context-dependent, shaped by environmental conditions and the biotic resistance of recipient communities^11–16^. Moreover, research in biological invasions rarely focuses on the role of biotic interactions, such as mutualisms, which are fundamental to the establishment of most plant species.

Emerging work suggests that mycorrhizal mutualisms in particular may be a key driver of non-native plant establishment^17,18^. Most terrestrial plants depend on mycorrhizal mutualisms, which comprise a few major mycorrhizal types^19,20^. Arbuscular-mycorrhizal associations are the most widespread, dominating in tropical and subtropical regions, whereas ectomycorrhizal associations are more common in cooler and drier forest biomes^21–23^. Beyond these dominant guilds, more specialized and phylogenetically restricted associations exist, such as orchid and ericoid mycorrhizae. Beyond association with certain mycorrhizal types, some plant species vary in their reliance on this mutualism (hereafter status), either being obligate or facultative-mycorrhizal. Facultative-mycorrhizal plant species are defined by being found both with and without their mycorrhizal partners^9,24,25^. Despite the fundamental importance of plant–mycorrhizal associations, the role of mycorrhizal strategy in invasion biology remains understudied, and it is unclear which mycorrhizal strategy is associated with non-native plant establishment across different biogeographic contexts.

Mycorrhizal plant species rely on the presence and viability of compatible fungal symbionts to establish^26^. The limited dispersal ability of certain mycorrhizal fungi, such as orchid and arbuscular-mycorrhizal fungi, can constrain host plant establishment^22,23,27–29^ and limit range expansion^30–32^, making the distribution and availability of symbionts a key factor influencing the success of non-native plants^33,34^. A well-documented example is the invasion of non-native *Pinus* species in the southern hemisphere, which is only possible through the co-invasion of ectomycorrhizal partners^35,36^. By contrast, non-mycorrhizal species bypass the need for fungal partners, whereas facultative-mycorrhizal species can adjust their strategy depending on local fungal symbiont availability^37^. Consequently, non-mycorrhizal and facultative-mycorrhizal strategies may be more optimal for non-native plant species. Indeed, regional- and global-scale studies have demonstrated that facultative-mycorrhizal non-native species have the highest naturalization success in mainland regions^24,25^.

A critical factor shaping the availability of fungal symbionts is the level of disturbance. Disturbances caused by land-use changes, such as agriculture, urban development or vegetation removal, can disrupt fungal networks and reduce symbiont abundance and diversity^27,38–40^. Disturbance events, such as fire and flooding, can have similar effects on soil microbial communities^41,42^, whether occurring naturally or as a result of human activity. Because disturbances reduce the reliability of mycorrhizal inoculum, they may amplify the relative advantage of plant species that can establish without mutualists (that is, non-mycorrhizal and facultative-mycorrhizal) over mycorrhizal species.

To date, it remains unclear whether non-mycorrhizal or facultative-mycorrhizal strategies are consistently advantageous to non-native species. The impacts of a given mycorrhizal strategy are likely to be context-dependent, shaped by biogeographical variation in climate, disturbance regimes and the availability and compatibility of fungal partners. Until now, few studies have directly compared the prevalence of mycorrhizal strategies between non-native and native species within the same communities at large spatial scales^10,16,43,44^. Instead, studies largely focus on the non-native flora in isolation from native flora, assessing the proportion of non-native species of each mycorrhizal type^24,25^. Importantly, this precludes testing whether the mycorrhizal strategy of non-natives differs from the dominant strategy of native plant species (that is, within a local community sharing biogeographical and environmental context). The few regional studies that do test for differences between the strategy of non-natives versus natives report contrasting results. For example, non-native species in California were found to be more non-mycorrhizal than natives^34^, whereas the opposite trend was observed in Great Britain^45^. To address these inconsistencies, our study evaluates mycorrhizal strategies on a global scale that accounts for both environmental variation and disturbances, offering a broader empirical basis for understanding the role of mycorrhizal associations in non-native plant establishment.

To achieve this, we investigate how biogeographic context and disturbance interact to influence the prevalence of mycorrhizal strategies in non-native plants globally. To do this, we combine vegetation plots from the sPlot global vegetation^46^ with information on the native status of plant species^47,48^ and their mycorrhizal type and status^49^. Specifically, we use statistical analyses to test the hypotheses that: (1) non-mycorrhizal and facultative-mycorrhizal species are over-represented among non-native compared with native plants, suggesting that these strategies confer advantages during establishment; (2) the prevalent mycorrhizal strategy of non-native plant species differs across biomes, driven by variation in environmental conditions and dominant mycorrhizal associations of native plant communities; and (3) the proportion of non-mycorrhizal and facultative-mycorrhizal non-native species increases with disturbance intensity, as altered environments reduce the availability of mycorrhizal partners. Although our observational approach identifies large-scale biogeographic patterns rather than direct causal mechanisms, these statistical associations provide compelling evidence for how mycorrhizal strategies relate to non-native plant establishment. Furthermore, they generate robust, testable hypotheses for future experimental studies to mechanistically disentangle how these fungal symbioses underpin the invasion process across global environments.

## Results and discussion

**Fig. 1:**
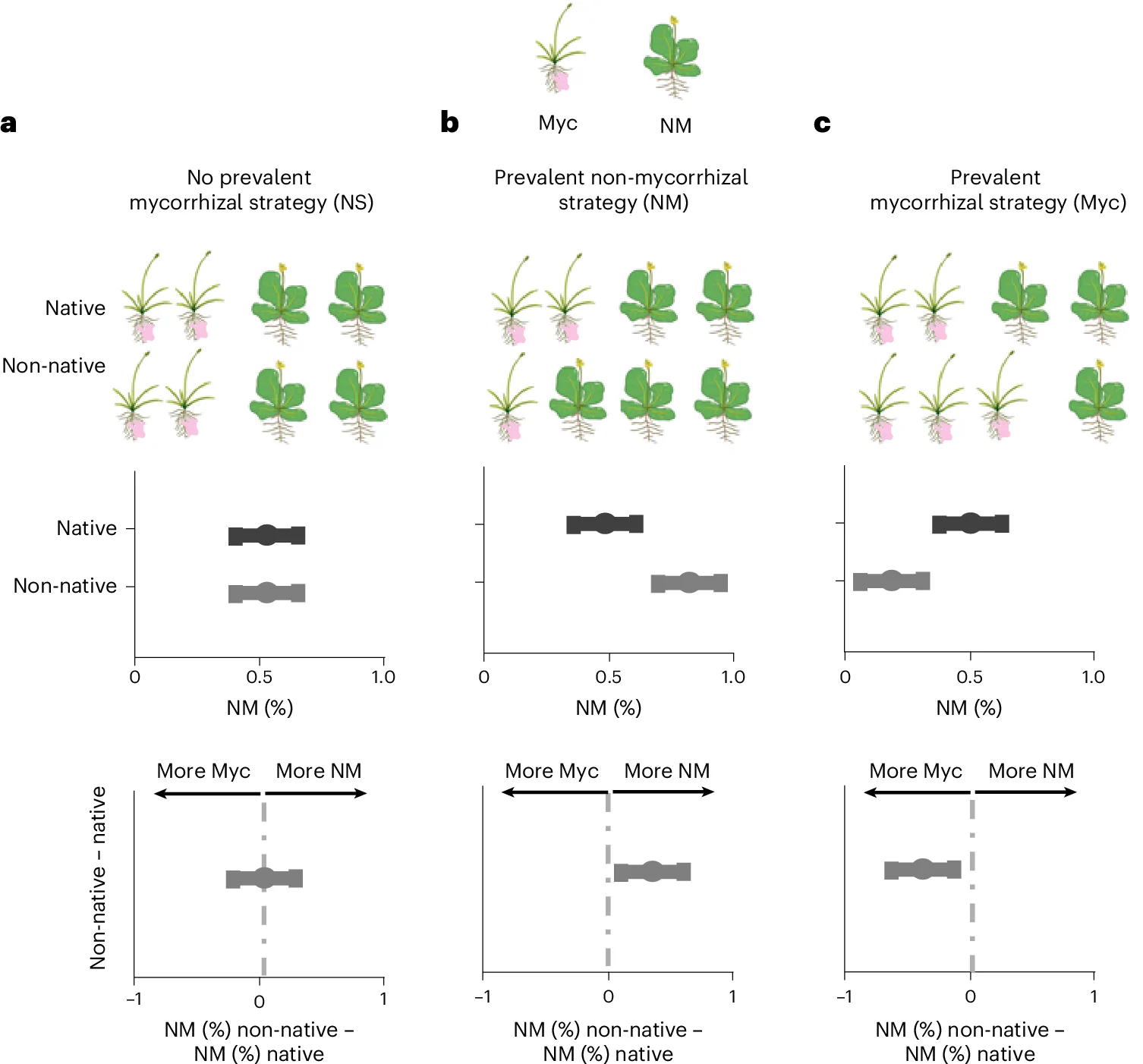
Conceptual framework for defining prevalent mycorrhizal strategies in non-native species. Schematic representation of three hypothetical scenarios comparing the prevalence of non-mycorrhizal (NM) versus mycorrhizal (Myc) strategies between native and non-native species within the same vegetation plot. **a**, No prevalent strategy: the proportion of NM species does not differ significantly between groups. **b**, Non-mycorrhizal strategy: NM species are significantly more prevalent in the non-native group, indicating that non-native plants adopt an NM strategy. **c**, Mycorrhizal strategy: NM species are significantly less prevalent (that is, Myc species are more frequent) in the non-native versus native groups, indicating that non-native species adopt a mycorrhizal strategy. Rows: hypothetical per-plot counts of Myc and NM species in native and non-native groups (top); model-estimated mean proportions of NM species with standard errors (s.e.) (middle); and the estimated difference in means (% NM_non-native_ − % NM_native_) with associated s.e. (bottom). NS, not significant. Credit: plant illustrations by Rachele Quaglino.

To evaluate how mycorrhizal strategies facilitate plant invasions, we compared the prevalence of non-mycorrhizal and facultative-mycorrhizal strategies between native and non-native species within vegetation plots. Using this approach, we captured global trends, biome-level variation and the influence of disturbances by first testing broad contrasts (for example, non-mycorrhizal versus all mycorrhizal types combined) followed by pairwise comparisons between individual mycorrhizal types (Fig. 1).

### Mycorrhizal strategy of non-native plants depends on biome

**Fig. 2:**
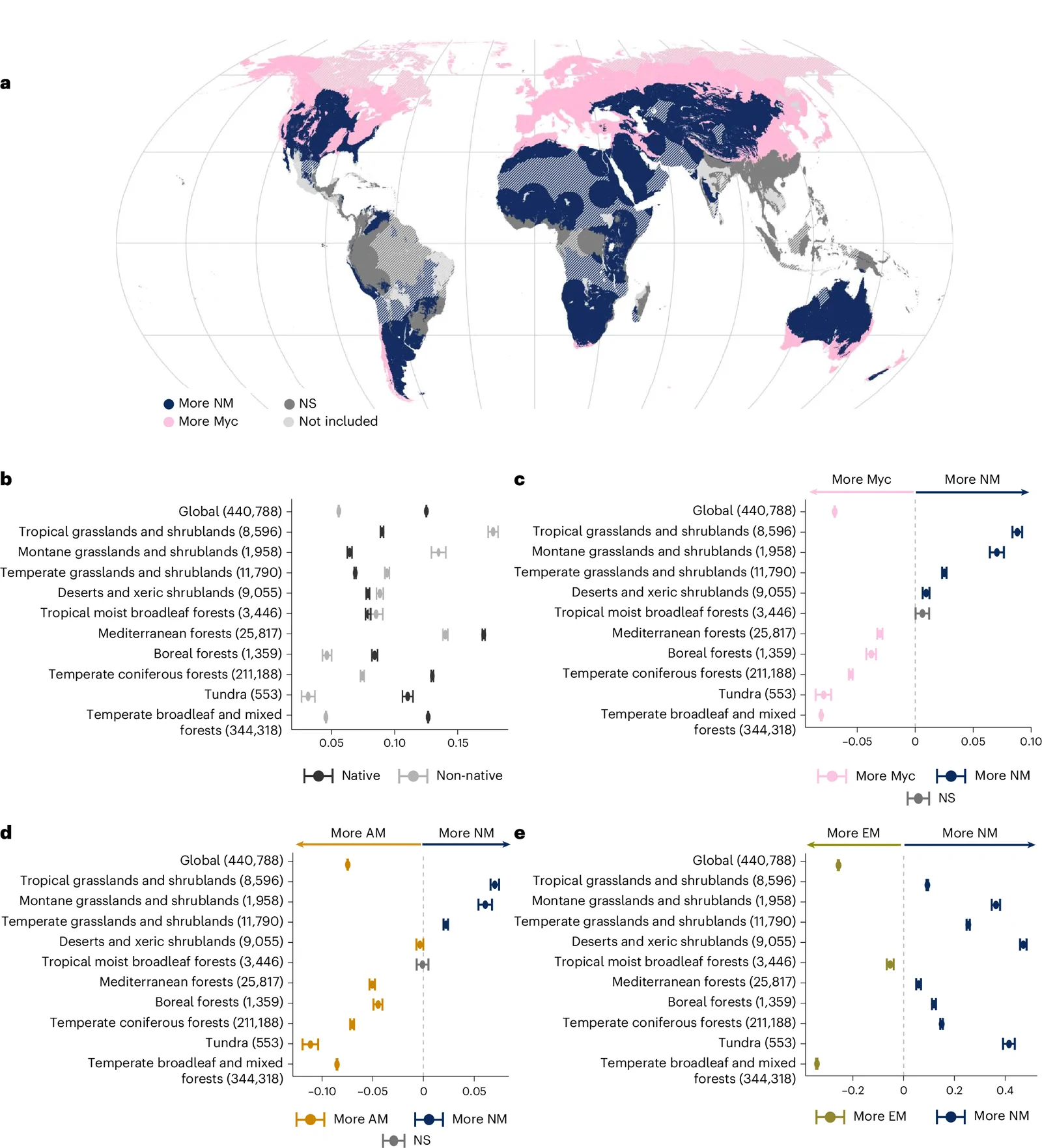
Relative prevalence of NM species in non-native versus native plants across biomes. **a**, Global distribution of prevalent non-native mycorrhizal strategies, comparing NM versus Myc; Myc includes counts of arbuscular-mycorrhizal (AM), ectomycorrhizal (EM) and facultative-mycorrhizal (FM) types. Biome colours represent the significantly dominant strategy of non-native relative to native plants (based on **c**). Light grey indicates biomes not analysed; hatching indicates areas >600 km from any sampling plot. **b**, Model-estimated mean proportions of NM species in native and non-native subgroups. **c**–**e**, Differences in mean NM proportions between non-native and native plants (non-native − native) for: NM versus Myc (**c**), NM versus AM (**d**) and NM versus EM (**e**). In **c**–**e**, positive values (dark blue) denote a prevalent NM strategy (higher NM prevalence in non-natives). Negative values denote a prevalent mycorrhizal strategy in non-natives: Myc (pink in **c**), AM (orange in **d**) or EM (green in **e**). Dark grey indicates no significant difference (*P* < 0.05). Error bars represent ±s.e. For full model outputs and contrasts, see Supplementary Table 3a–c. Map in **a** created with R package terra^99^. Source data

At a global scale, we found that there is a higher probability that non-native species are mycorrhizal compared with native species (Fig. 2b,c), suggesting that mycorrhizal associations may confer an advantage for non-native species. However, this pattern varied strongly across biomes and across different ranges of environmental conditions (Extended Data Fig. 5). The global pattern was recovered in most forested temperate regions, where non-native species are predicted to be more mycorrhizal than native species are. However, in most grasslands and drylands, we found a higher probability that non-native species are non-mycorrhizal compared with native species (Fig. 2a–c and Extended Data Fig. 6a–c). These biome-level results suggest that the mycorrhizal strategy of non-natives is context-dependent.

Beyond the broad mycorrhizal strategy of non-native species (mycorrhizal), we found that the specific mycorrhizal strategy (type) varied across biomes. Mycorrhizal species were over-represented in non-native relative to native species in temperate moist broadleaf forests, Mediterranean forests, temperate coniferous forests, boreal forests and tundra biomes, which are generally dominated by ectomycorrhizal plants. In these regions, we found that non-native species were more likely to be arbuscular-mycorrhizal than non-mycorrhizal relative to native species. Further, non-native plant species were more likely to be non-mycorrhizal than ectomycorrhizal relative to native species in these regions, except for temperate broadleaf and mixed forests, where ectomycorrhizal were over-represented. This suggests that, where non-native plants are more likely to be mycorrhizal, they are also more likely to be arbuscular-mycorrhizal, except for in temperate broadleaf and mixed forests, where non-natives are predicted to associate more with ectomycorrhizal compared with natives. The increased probability of non-native species to be mycorrhizal and primarily arbuscular-mycorrhizal in these biomes aligns with the more ruderal state of mostly arbuscular-mycorrhizal understory in these systems. That is, the species that are more likely to invade are arbuscular-mycorrhizal herbs. Where ectomycorrhizal strategies are over-represented compared with non-mycorrhizal, these species may promote success in forested biomes where they are ecologically dominant within the native community^23^.

In contrast, we found a higher probability that non-native species are non-mycorrhizal compared with native species in grassland biomes, which are generally dominated by arbuscular-mycorrhizal plants^50^. The most striking example of this pattern was observed in tropical grasslands and shrublands, with models predicting that non-native species comprised 17.8% non-mycorrhizal, nearly double their proportion among natives (9.0%) (Fig. 2). In these biomes, early-invading non-mycorrhizal species may reduce fungal abundance and diversity, thereby weakening native plant recruitment and facilitating further non-mycorrhizal invasions^51–55^. This highlights how non-mycorrhizal success may be contingent upon the mycorrhizal type of the native community and the disruption of local fungal-mediated feedbacks. The over-representation of non-mycorrhizal non-native species in these biomes may also reflect fundamental differences in soil nutrient cycling. These ecosystems typically have rapid nutrient turnover rates where arbuscular-mycorrhizal fungi facilitate phosphorus acquisition^51,56^. However, disturbed versions of these systems—where invasions are most prone to occur—may have a phosphorus surplus from anthropogenic inputs, causing non-mycorrhizal species to gain an advantage by bypassing the carbon cost of the arbuscular-mycorrhizal symbiosis when phosphorus is not limiting^57^.

### Mycorrhizal strategy of non-native plants depends on disturbance intensity and type

**Fig. 3:**
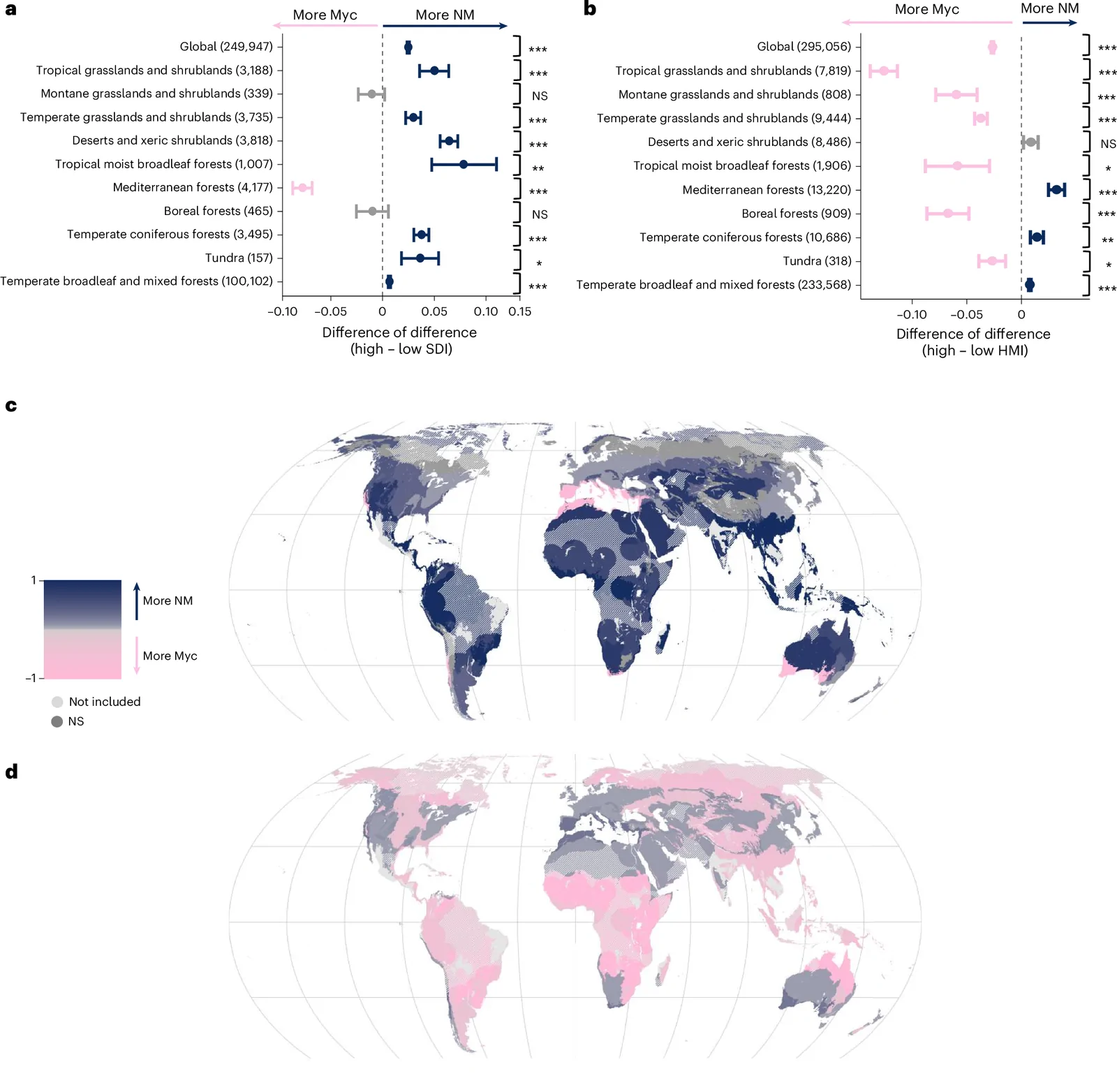
Effect of disturbance intensity and type on the proportion of non-mycorrhizal species. We assessed how high disturbance shifts mycorrhizal types using two metrics: the SDI, a proxy for generalized natural and human-induced disturbance and the HMI, which reflects human pressure. **a**,**b**, Biome-level differences in model-estimated mean proportions of NM species between high and low disturbance for SDI (**a**) and HMI (**b**). Positive values (blue) indicate a shift towards an NM strategy under high disturbance; negative values (pink) indicate a shift towards a Myc strategy (including AM, EM and FM species). Asterisks denote significant pairwise contrasts (*P* < 0.05); error bars represent ±s.e. **c**,**d**, Global maps showing biome-level shifts in non-native mycorrhizal strategy under high SDI (**c**) and high HMI (**d**). Biomes are coloured according to the shift in the relative prevalence of NM species (the difference in NM proportions between high and low disturbance). The colour gradient represents the square-root scaled difference, ranging from dark blue (value of 1; maximum shift towards NM strategy) to dark pink (value of −1; maximum shift towards Myc strategy). Grey indicates biomes with no significant shift; light grey indicates biomes not included in the analysis. Hatched areas indicate regions without sampling plots within a 600-km radius. Maps in **c** and **d** created with R package terra^99^. Source data

**Extended Data Fig. 1:**
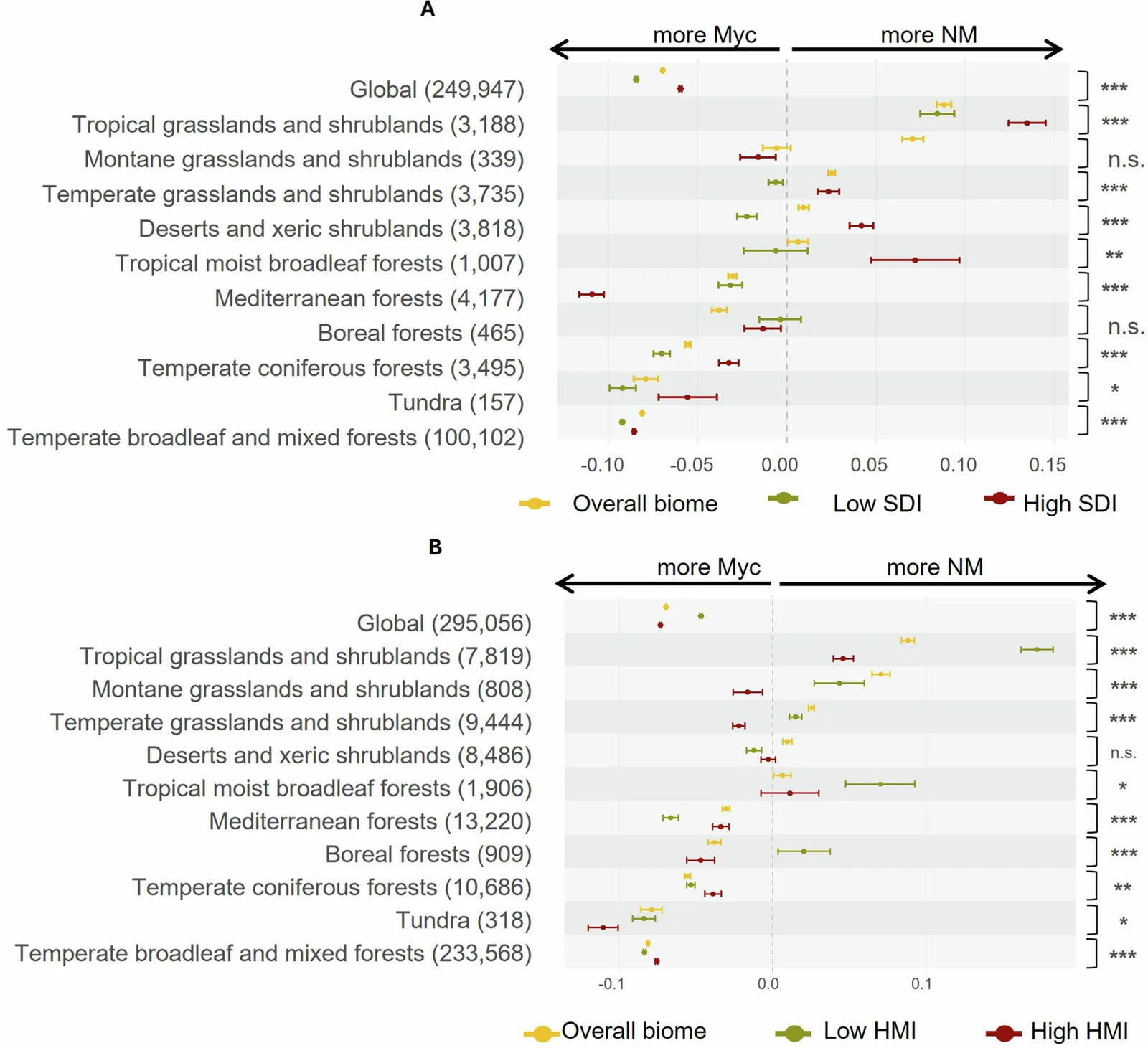
Effect of disturbance intensity and type on the prevalence of non-mycorrhizal (NM) species. (**A**, **B**) Biome-level differences in model-estimated mean proportions of NM species between non-native and native plants across two disturbance metrics: (**A**) Structural Disturbance Index (SDI) and (**B**) Human Modification Index (HMI). Bars represent estimated marginal means for the overall biome average (yellow), low disturbance (green), and high disturbance (red). Asterisks to the right of the bars denote significant differences between high and low disturbance conditions based on pairwise contrasts (p < 0.05). Error bars represent ± SE. See Supplementary Tables S3 A3–A6 for comprehensive model statistics and contrasts. Source data

We used two distinct metrics to assess the effect of disturbance on the mycorrhizal strategy of non-native plants: the structural disturbance index (SDI), which is a proxy for generalized disturbance as it accounts for the intensity and frequency of natural and human-caused events, such as fires and logging, and the global human modification index (HMI), which reflects stable human pressure such as urbanization and infrastructure. We found that high generalized disturbance increases the probability of non-native species being non-mycorrhizal compared with natives at the global level and in most biomes (Fig. 3a,c and Extended Data Fig. 1a). This pattern was especially pronounced in tropical grasslands and shrublands, deserts and xeric shrublands and tropical moist broadleaf forests. These biomes are typically dominated by arbuscular-mycorrhizal associations^50^, yet are increasingly subject to natural (for example, fire and grazing) and anthropogenic (for example, land conversion and deforestation) disturbances^58–61^ (Extended Data Fig. 7). Since arbuscular-mycorrhizal fungi have limited dispersal capacity^62^, such disturbances can degrade arbuscular-mycorrhizal fungal inoculum, reducing establishment opportunities for arbuscular-mycorrhizal-dependent species. Consequently, non-mycorrhizal species—often ruderal, disturbance-adapted and unconstrained by symbiont dependence—can rapidly exploit these conditions^63,64^. This helps to explain the consistent rise of non-mycorrhizal strategies among non-natives in highly disturbed arbuscular-mycorrhizal systems^52,54,65^. In contrast, the opposite pattern was found in Mediterranean forests: increased generalized disturbance was associated with higher proportions of mycorrhizal species among non-natives (Fig. 3a,c). Unlike arbuscular-mycorrhizal-dominated systems, these forests often host ectomycorrhizal or mixed arbuscular-ectomycorrhizal long-lived woody perennials^50,66^. These systems may thus retain functional mycorrhizal associations even under disturbance because of the the resilience of trees and associated ectomycorrhizal fungi, which produce resistant propagules, disperse more effectively and can persist independently of hosts^20,67,68^. Specifically, the frequent fire and drought of Mediterranean ecosystems may have selected for ectomycorrhizal spore banks that are more resilient than those in boreal or temperate forests, where disturbance leads to severe fungal biomass decline^69–71^.

The HMI, reflecting more stable human pressure such as urbanization and infrastructure, was generally associated with increased prevalence of mycorrhizal species among non-natives (Fig. 3b,d and Extended Data Fig. 1b). These results align with results by ref. ^72^, which showed that roadside disturbance promoted mycorrhizal species. Specifically, increasing human pressure was associated with a higher proportion of mycorrhizal non-natives in grasslands, tropical moist broadleaf forests, boreal forests and tundra, biomes that were more likely to be non-mycorrhizal under high generalized disturbance. By contrast, for Mediterranean and temperate forest biomes (both coniferous and broadleaf), increased human pressure was associated with higher proportions of non-mycorrhizal non-natives. These contrasting patterns between generalized disturbance and human pressure underscore the need to consider several disturbance types when assessing invasion dynamics. Independence from mycorrhizal fungi may confer an advantage in systems with degraded fungal communities or altered nutrient regimes, where non-mycorrhizal species can bypass the carbon costs of symbiosis^57,73,74^. By contrast, associations with mycorrhizal fungi may confer an advantage to non-native species in less frequently disturbed, human-modified systems where fungal partners persist or are actively spread through human activities (for example, mycorrhizal crops and ornamental species).

### Facultative-mycorrhizal strategy does not confer an advantage to non-native establishment

**Fig. 4:**
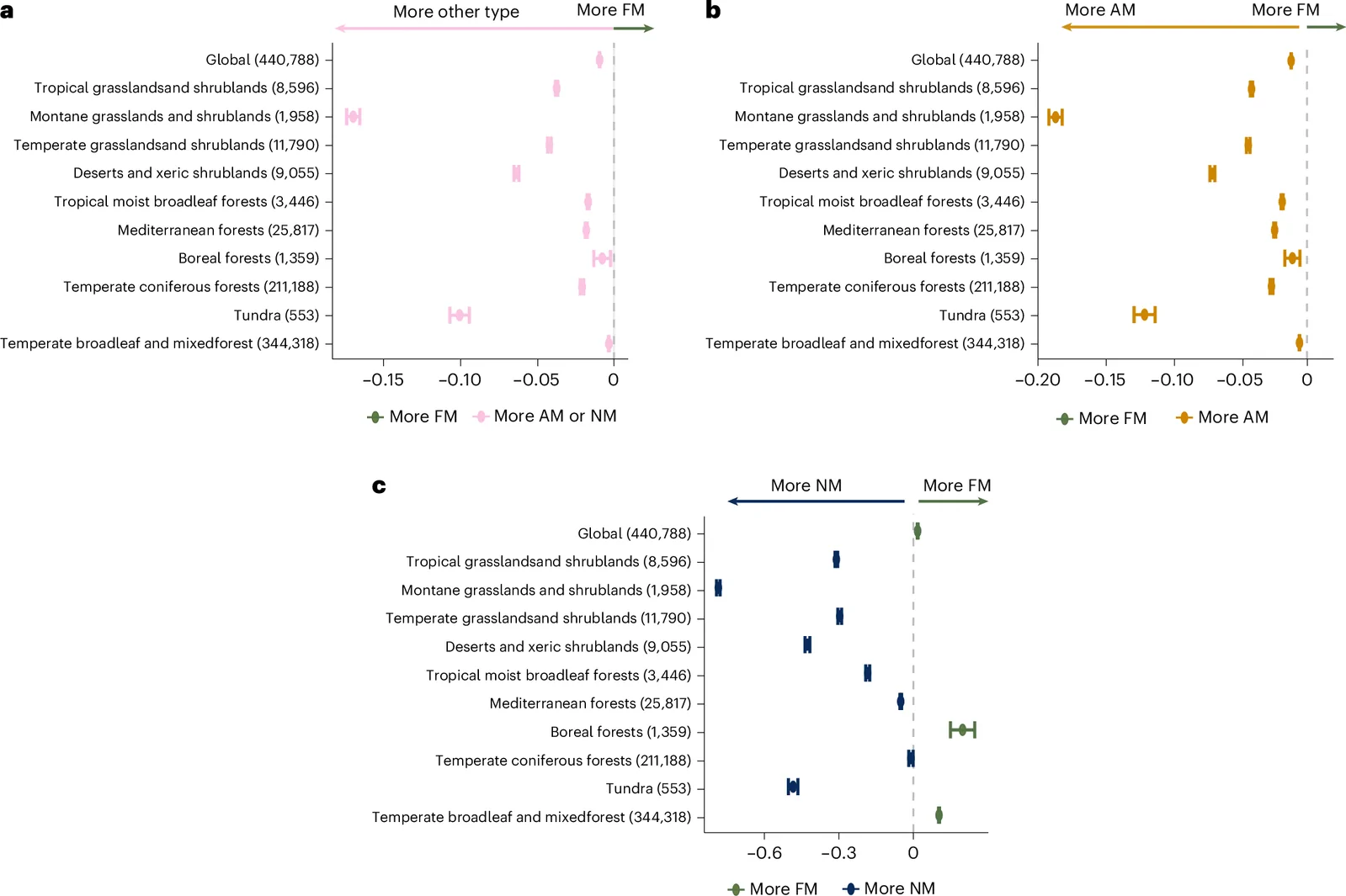
Relative prevalence of FM species in non-native versus native plants across biomes. Bars represent the difference in model-estimated mean proportions of FM species between non-native and native plants. **a**–**c**, Comparisons are shown for FM versus all other types (AM + NM) (**a**), FM versus AM species (**b**) and FM versus NM species (**c**). Positive values (dark green) indicate that FM species are more prevalent among non-natives, suggesting a non-native preference for the FM strategy. Negative values indicate a higher non-native prevalence of the alternative group: AM + NM (pink) (**a**), AM (orange) (**b**) or NM (light green) (**c**). Error bars represent ±s.e. Sample sizes (*n* plots) are indicated adjacent to biome names. For comprehensive model outputs and pairwise contrasts, see Supplementary Table 2a–c. Source data

**Extended Data Fig. 2:**
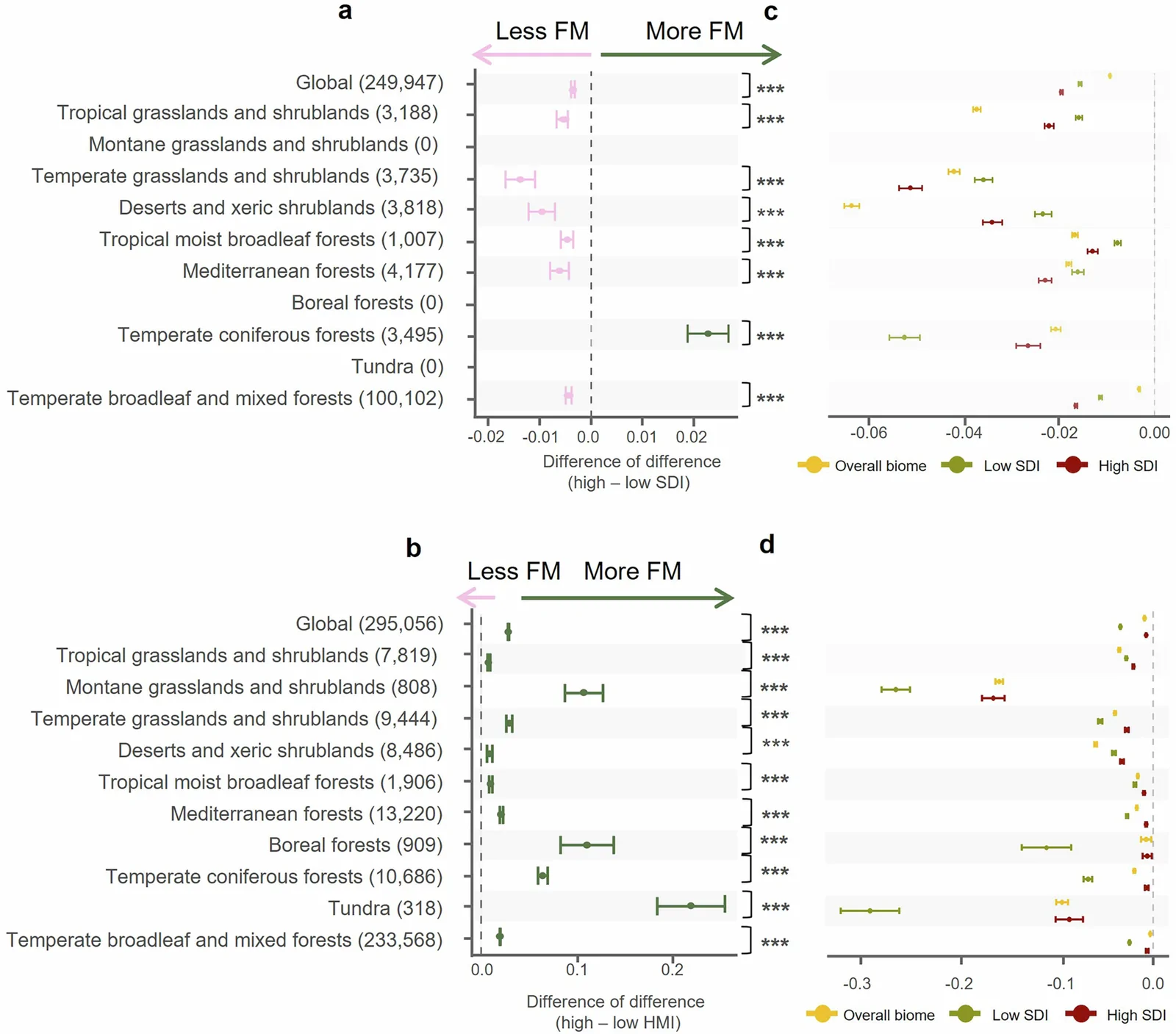
Effects of disturbance intensity and type on the prevalence of facultative-mycorrhizal (FM) species. (**A**, **B**) Biome-level differences in model-estimated mean proportions of FM species between high and low disturbance for SDI (**A**) and HMI (**B**). Positive values (green) indicate an increase in FM prevalence under high disturbance; negative values (pink) indicate a shift toward alternative strategies (AM or NM). Asterisks denote significant pairwise contrasts between high and low disturbance conditions (p < 0.05). (**C**, **D**) Comparison of FM proportions between non-native and native plants (non-native - native) across biomes under varying disturbance levels. Bars represent differences under low disturbance (green) and high disturbance (red) for SDI (**C**) and HMI (**D**). Yellow bars indicate the baseline per biome. Error bars represent ±SE. See Supplementary Tables S2 A3–A6 for full model statistics and contrasts. Source data

The facultative-mycorrhizal strategy has been identified as a key driver of naturalization success, with facultative-mycorrhizal species typically establishing in more regions and over larger geographic areas than obligate or non-mycorrhizal species^24,25,75^. This suggests that the flexibility to switch between mycorrhizal states provides the broad environmental niche necessary for global range expansion. However, our plot-level analyses show that facultative-mycorrhizal species are consistently under-represented among non-native compared with native plants (Fig. 4a,c). Furthermore, we found that disturbance did not shift the qualitative status of the facultative-mycorrhizal strategy; facultative-mycorrhizal species remained consistently under-represented among non-natives relative to natives across low and high disturbance levels (Extended Data Figs. 1 and 6). Nonetheless, disturbance increased the relative proportion of facultative-mycorrhizal among non-natives. Specifically, high human pressure was associated with an increased proportion of facultative-mycorrhizal species across all biomes (Extended Data Fig. 2a), and a similar trend was observed for generalized disturbance in temperate coniferous forests (Extended Data Fig. 2b). These patterns may reflect ecological limitations of the facultative-mycorrhizal strategy during the invasion process. In undisturbed or mycorrhizal-rich systems, facultative-mycorrhizal species may be outcompeted by obligate arbuscular-mycorrhizal plants that form more efficient or specialized fungal associations^65^. In highly disturbed environments, facultative-mycorrhizal species may lack the stress tolerance or rapid resource acquisition strategies of non-mycorrhizal species. Contrary to expectations based on the broad ecological flexibility and wide ranges typically associated with facultative-mycorrhizal species^24,25,75,76^, we found facultative-mycorrhizal species to be under-represented among non-natives at the plot level. This suggests that the facultative-mycorrhizal strategy does not confer a consistent advantage in invasion. By directly benchmarking non-native strategies against co-occurring natives, we demonstrate that local invasion success is not merely a product of inherent ecological plasticity, but rather depends on how the strategy of a species diverges from the established native baseline.

### Limitations and future directions

**Extended Data Fig. 3:**
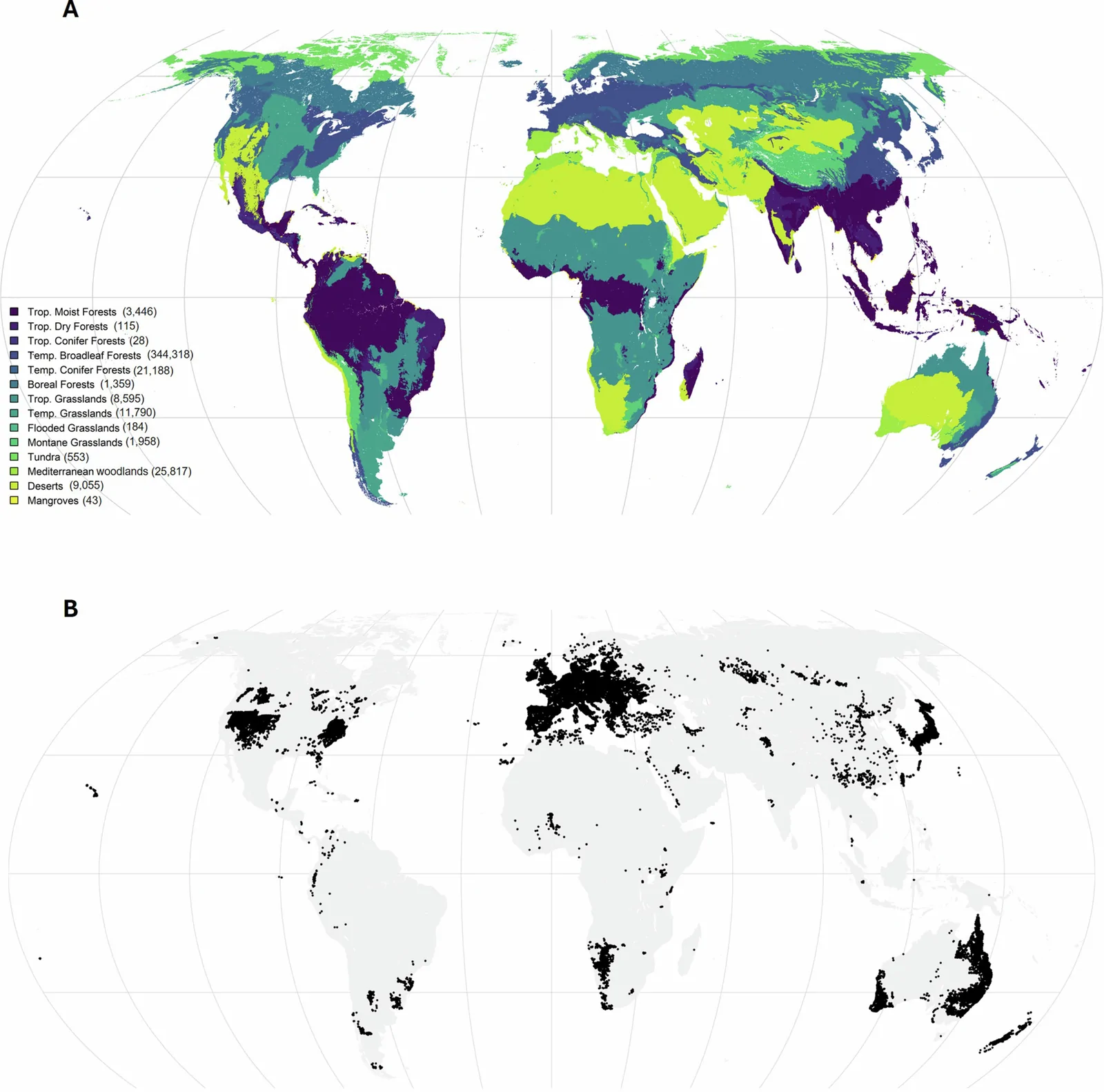
Geographic distribution of biomes and sampling intensity. (**A**) Global distribution of biomes as defined by Dinerstein et al. (2017). Colors correspond to the biomes analyzed, with the total number of vegetation plots indicated for each. (**B**) Geographic locations of the 440,788 vegetation plots (black dots) included in this study. Maps in **a** and **b** created with terra^99^.

**Extended Data Fig. 4:**
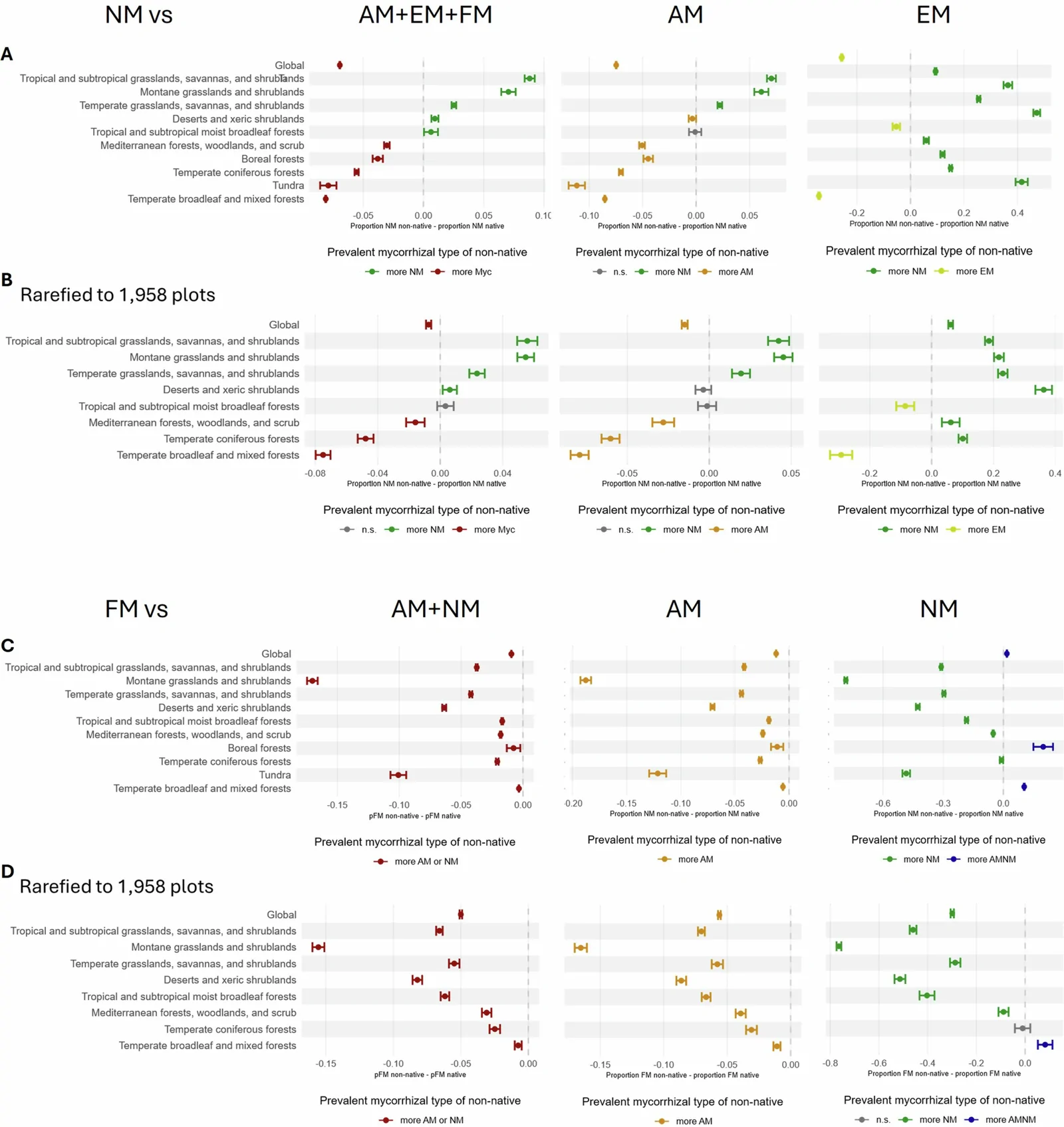
Sensitivity analysis comparing non-rarefied and rarefied datasets. Estimated differences in the mean proportion of (**A**, **B**) non-mycorrhizal (NM) and (**C**, **D**) facultative-mycorrhizal (FM) species between non-native and native plants (non-native - native). Left panels (**A**, **C**) show results using the full dataset (*n* = 440,788 plots); right panels (**B**, **D**) show results using the rarefied dataset, where all biomes were subsampled to match the montane grassland biome (*n* = 1,958 plots per biome). Positive values indicate a significantly higher prevalence of the focal strategy in non-natives: NM (green in **A**, **B**) or FM (blue in **C**, **D**). Negative values indicate a significantly higher prevalence of the alternative group in non-natives: Myc (other types combined: AM+EM+FM) (red), arbuscular mycorrhizal (AM) (orange), or ectomycorrhizal (EM) (yellow). Dark grey indicates no significant difference. Error bars represent ± SE. See Supplementary Tables S3 and S4 for detailed model outputs and contrasts. Source data

Although our findings provide a broad framework for understanding the mycorrhizal strategies of non-native plants, they should be interpreted in light of several important limitations. Although sPlot represents the largest available plant co-occurrence (ref. ^46^ and Damasceno et al., in preparation), certain regions of the world are considerably undersampled relative to others (Extended Data Fig. 3b). Rarefied results closely mirrored those of the full dataset, with global and biome-level trends remaining consistent (Extended Data Fig. 4 and Supplementary Tables 3 and 4), suggesting that the main patterns reported are robust, even with regional sampling bias. Nonetheless, only increased investment in sampling under-represented regions can fully address these limitations and this remains an active goal of the sPlot community^77^. Another limitation is that the analyses presented were conducted only on invaded plots. However, non-native establishments may alter the mycorrhizal-type composition of the native community. When we accounted for this effect at the biome level, our results remained largely consistent (Supplementary Table 7). Although this assessment aligns with our major conclusions, this highlights the value of plot resampling to compare native communities in uninvaded and subsequently invaded locations. Finally, although we used the most up-to-date empirical data from the FungalRoot database, associated mycorrhizal information remains incomplete, covering only a small fraction of the global flora^78^, with marked geographic and taxonomic biases. In addition, where species-level information was unavailable, mycorrhizal status and type were inferred from genus-level assignments, an approach that enables global coverage but introduces uncertainty as a result of documented within-genus variability in mycorrhizal associations^78,79^. Further, these assignments may reflect both genuine intraspecific variability and limitations in sampling intensity or diagnostic resolution (for example, for facultative-mycorrhizal), which can introduce uncertainty in mycorrhizal classification. Beyond taxonomic coverage, an ecological limitation of our analysis is that these assignments do not account for quantitative differences in mycorrhizal reliance and growth response. Because the fitness benefits of mycorrhizal associations are highly context-dependent, species with high reliance may face different establishment barriers compared with those with low or facultative reliance, particularly under the varying disturbance regimes and environmental conditions. This underscores the need for more species- and population-level assessments of mycorrhizal type, colonization rates^80^ and growth benefit^81^. Finally, our study is correlational in nature and experiments will be needed to mechanistically test for invasibility of different mycorrhizal types under different biotic and environmental conditions. Despite these limitations, this study draws on the largest global plant co-occurrence dataset, uses best practices in mycorrhizal classification and uses two global disturbance metrics to present the most comprehensive analysis of the mycorrhizal strategy of non-native plant species worldwide.

Our results show that the mycorrhizal strategy of non-native plants is highly context-dependent, shaped by biome and disturbance regimes. In many arbuscular-mycorrhizal-dominated systems, species that rely on the non-mycorrhizal strategy are over-represented among non-native compared with native species occurring in the same community. By contrast, ectomycorrhizal-dominated systems show mixed mycorrhizal strategies of non-native plants. Moreover, our results show that although disturbance is a key predictor of mycorrhizal strategy, the direction of this effect hinges on the nature of the disturbance. Specifically, generalized disturbance increases the proportion of non-mycorrhizal, whereas human pressure favours mycorrhizal non-native species. These results suggest distinct underlying mechanisms: whereas sustained and frequent disturbance probably depletes fungal inoculum, stable anthropogenic pressures appear to preserve, or even facilitate, mutualisms between fungi and non-native plant species. Finally, facultative-mycorrhizal species, often assumed to be ecologically advantageous for invasion, are under-represented among non-native species and at all disturbance levels, refuting the longstanding idea that symbiotic flexibility promotes the establishment of non-native species. Ultimately, these findings shed light on the joint role of biome and disturbance on which mycorrhizal strategies are more likely to establish and present invasion risks. Together, this information contributes to refining invasion risk predictions and informing more targeted, effective management strategies based on mycorrhizal strategy and environmental context.

## Methods

### Census data and native status assignment

We used vegetation-plot data from the sPlot database (v.4.0; ref. ^46^ and Damasceno et al. in preparation), a global compilation of 2.5 million georeferenced plot surveys conducted from 1888 to 2022 across all major biomes of the world (plot size from 1 to 1,000 m^2^). Plant species at each vegetation plot were assigned a native status (native or non-native) based on consensus classifications from the Global Naturalized Alien Flora (GloNAF)^47^ and the Plants of the World Online (POWO) database^48^.The GloNAF database contains comprehensive, spatially explicit data on the naturalization status of over 10,000 plant species across 1,029 geopolitical regions worldwide. The POWO database provides information on the native distribution of vascular plant species, covering more than 1.4 million plant names. We linked species occurrence data from sPlot to GloNAF regions by matching species names with their geographic coordinates and then extracting the corresponding GloNAF region codes using Google Earth Engine^82^. The same procedure was applied to assign species native status from the POWO database. To minimize uncertainty in native status classification, we excluded plots in which any occurring species had conflicting native status assignments between the two databases. For plots resampled several times, we only kept the most recent census. On the basis of this filtering, we retained 65,438 plant species across 2,231,767 vegetation plots (from 2,536,019 initial plot observations).

### Mycorrhizal type and status assignment

Mycorrhizal strategies (either type or status) were assigned on the basis of data from the FungalRoot database^49^. Plant mycorrhizal types, that is, arbuscular-mycorrhizal, ectomycorrhizal, dual arbuscular-mycorrhizal and ectomycorrhizal mycorrhizal and non-mycorrhizal, were first assigned at the species level. Given the limited availability of species-level information for many plant taxa^78^, we assigned mycorrhizal type at the genus level when species-level data were missing. This approach, based on all available empirical records for species within each genus, assigns the most frequent mycorrhizal type and status as representative for that genus^49^. Although extrapolation to the genus level involves assumptions, it is widely used when species coverage is incomplete and provides a substantial gain in trait data coverage, with assignments considered as working hypotheses until more comprehensive species-level data become available^79,83^. Although ecologically relevant, orchid and ericoid mycorrhizal types were excluded from our analyses because they represent a very small percentage of the global flora in the sPlot database (sPlot species assigned: orchid mycorrhizal of 0.02%) or were absent (ericoid mycorrhizal of 0%). To ensure sufficient mycorrhizal data at each plot included in the analyses, only plots with at least 80% of species assigned to a mycorrhizal status were considered, retaining 62,145 plant species and 1,867,058 vegetation plots. This mycorrhizal-type information was then used to assign mycorrhizal status as mycorrhizal (arbuscular-mycorrhizal and ectomycorrhizal), non-mycorrhizal or facultative-mycorrhizal. Facultative-mycorrhizal plants can switch between ectomycorrhizal and non-mycorrhizal strategy. Facultative-ectomycorrhizal species were not present in the database; dual arbuscular-ectomycorrhizal plants were too few (0.004%) and thus excluded from the analyses. For each vegetation plot, we calculated the number of species in each mycorrhizal type (obligate mycorrhizal, facultative-mycorrhizal and non-mycorrhizal) and mycorrhizal status (arbuscular-mycorrhizal, ectomycorrhizal, facultative-mycorrhizal and non-mycorrhizal), separately for native and non-native plants. Finally, we retained only plots where non-native plant species occur (non-native count >0). This resulted in a final dataset that included 440,788 vegetation plots with 39,731 plant species from 14 biomes, sampled between 1899 and 2022 (Extended Data Fig. 3).

### Biome assignment and environmental variables

**Extended Data Fig. 5:**
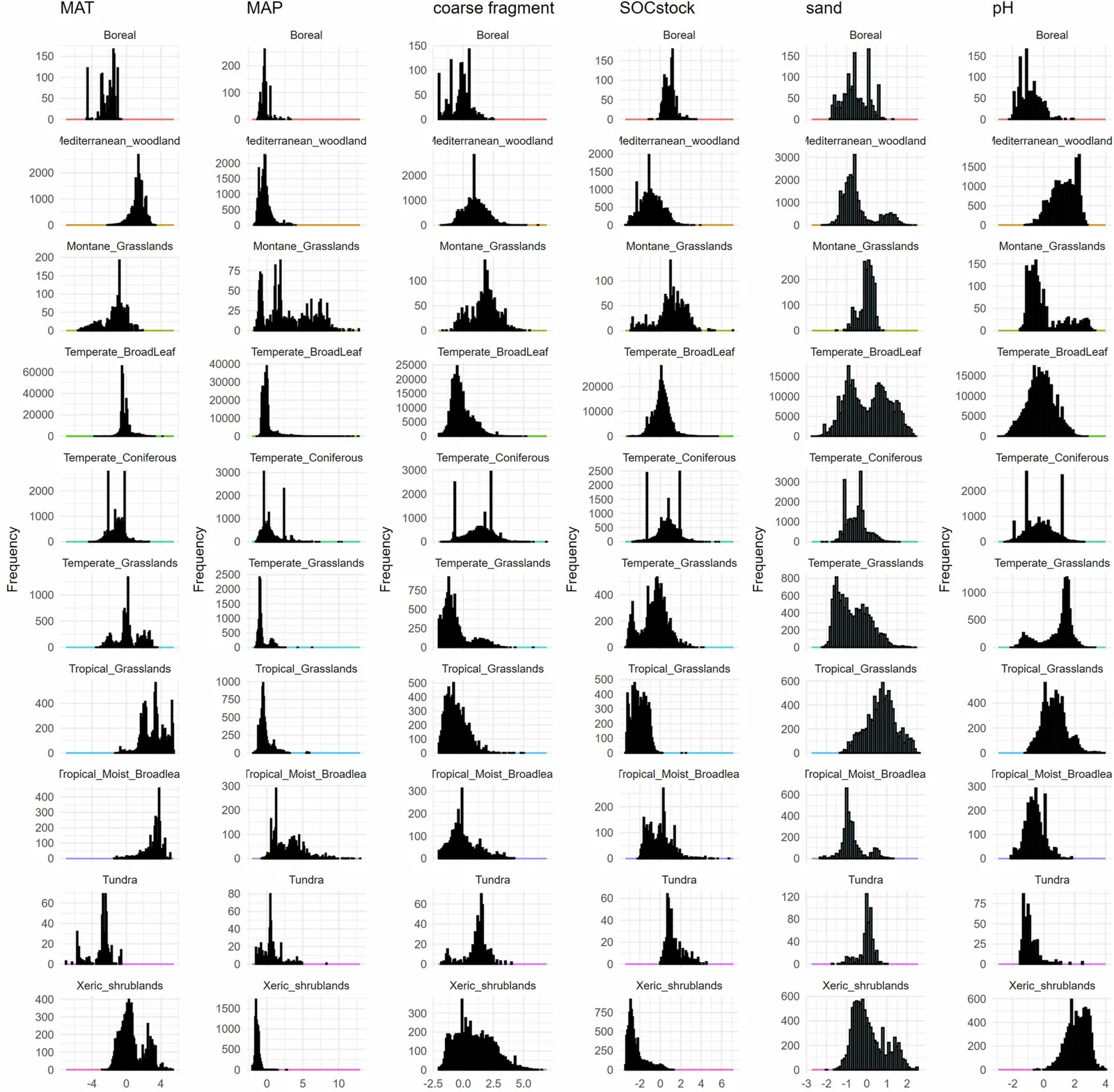
Model sensitivity analysis including soil nutrient covariates. Density plots illustrate the distribution of covariates included in the global models. The x-axes represent centered and scaled values for each environmental variable; y-axes indicate the density of vegetation plots. Facets correspond to the biomes analyzed in the study. Source data

**Extended Data Fig. 6:**
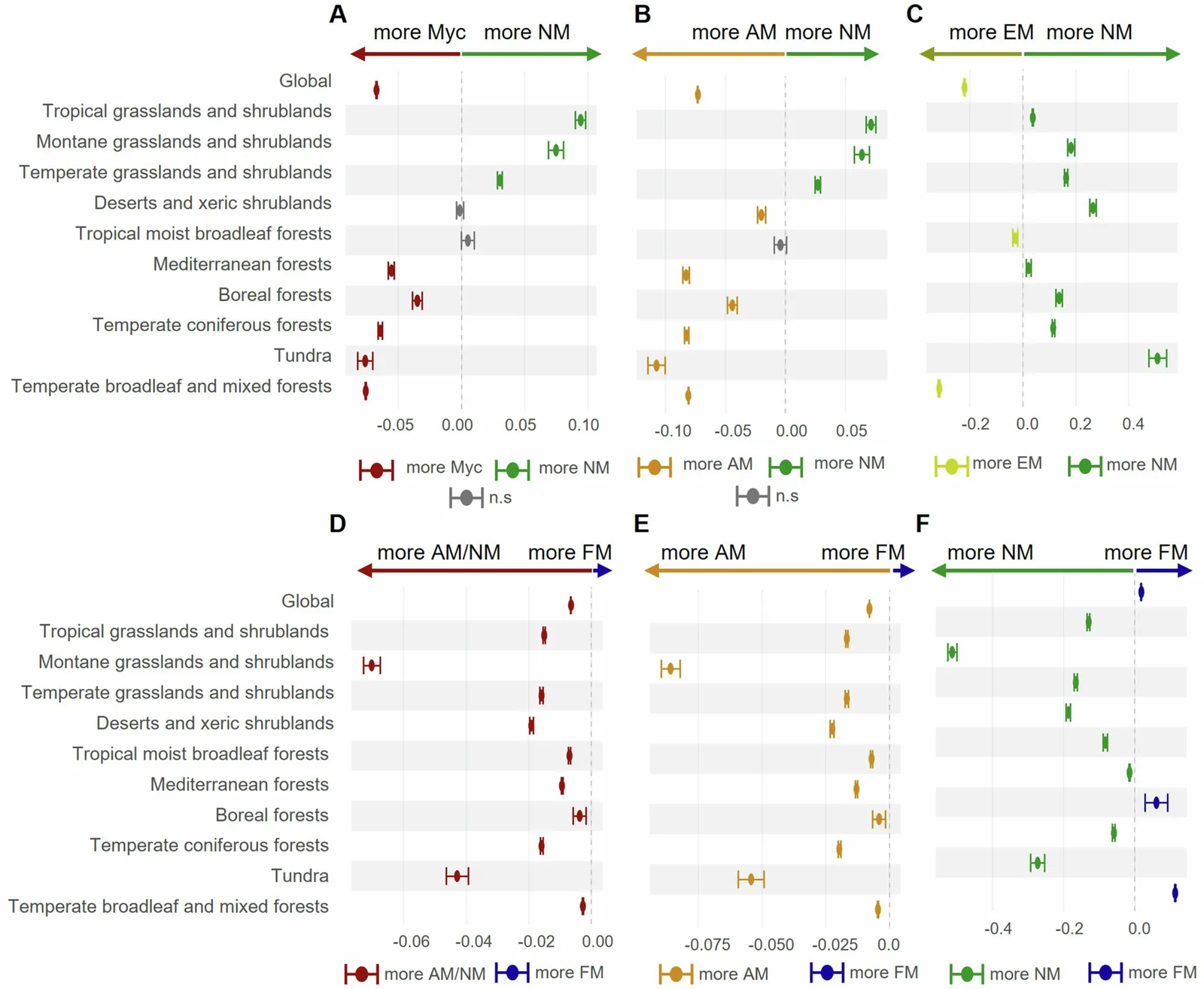
Distribution of environmental and soil variables across biomes. Estimated differences in the mean proportion of (**A**–**C**) non-mycorrhizal (NM) and (**D**–**F**) facultative-mycorrhizal (FM) species between non-native and native plants (non-native - native) across biomes. Models include nitrogen and phosphorus concentrations as covariates (n = 385,105). (**A**, **D**) Focal group vs. all other mycorrhizal types combined; (**B**, **E**) focal group vs. arbuscular mycorrhizal type (AM); and (**C**, **F**) focal group vs. ectomycorrhizal (EM) type.Positive values indicate a significantly higher prevalence of the focal strategy in non-natives: NM (green in **A**–**C**) or FM (blue in **D**–**F**). Negative values indicate a significantly higher prevalence of the alternative group in non-natives: all other types (red), AM (orange), or EM (yellow). Dark grey indicates non-significant differences. Error bars represent ± SE. See Supplementary Table S5 for full model outputs.

Vegetation plots were assigned to biomes according to the classification of ref. ^84^. To characterize environmental conditions at each plot location, we integrated high-resolution spatial data on climate and soil properties. Climatic variables were derived from the CHELSA dataset^85^, which provides fine-scale (~1 km^2^) temperature and precipitation layers designed for ecological and biogeographical research. Soil properties were obtained from the SoilGrids database^86^, a global resource that provides standardized estimates of soil attributes at 250-m resolution. All environmental layers were harmonized to a spatial resolution of 30 arcsec (~1 km^2^ at the equator) to ensure consistency across datasets^87^. To reduce redundancy and avoid multicollinearity among predictor variables, we selected a subset of environmental variables with low collinearity (correlation values <0.6)^88^. The final set of predictors included mean annual temperature (mean yearly temperature averaged across years) and mean annual precipitation (total yearly precipitation averaged across years) to reflect the variation in broad climatic trends, along with several soil variables measured at a depth of 5 cm: volumetric fraction of coarse soil fragments (cm^3^ dm^−^^3^), proportion of sand particles (g kg^−1^), soil organic carbon stock (dg kg^−1^) and soil pH. To evaluate the influence of soil nutrients, we conducted a sensitivity analysis incorporating soil nitrogen^86^ and phosphorus^89^. Owing to high spatial uncertainty in global nutrient rasters, these were analysed in separate models to avoid overinterpreting their causal weight. Results remained qualitatively consistent with our primary findings, demonstrating that observed patterns are robust to the inclusion of soil nutrient gradients (Extended Data Fig. 6 and Supplementary Table 4), with minor variations in xeric deserts probably due to reduced sample sizes (387,593 plots). All variables were standardized (scaled and centred) before their inclusion in statistical models to ensure comparability (see Extended Data Fig. 5 for distribution of environmental values across biomes).

### Disturbance and human pressure metrics

**Extended Data Fig. 7:**
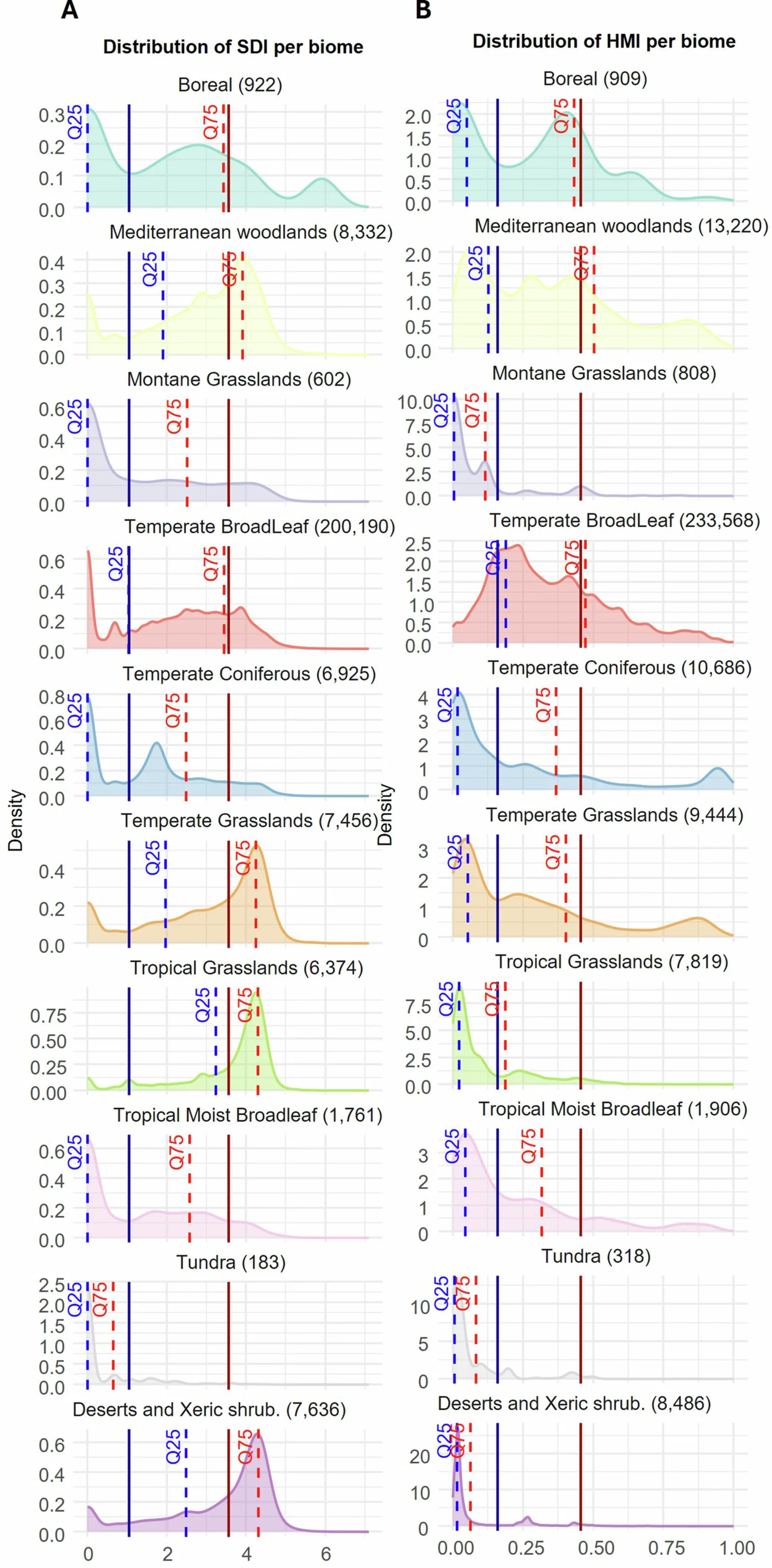
Distribution of disturbance metrics across biomes. Density plots showing the distribution of (**A**) Structural Disturbance Index (SDI) and (**B**) Human Modification Index (HMI) across the analyzed biomes. Y-axes are free-scaled to highlight the internal distributional patterns of each biome. Solid vertical lines indicate the global 25^th^ (blue) and 75^th^ (red) percentiles of the respective disturbance metrics. Dashed vertical lines represent the 25^th^ (blue) and 75^th^ (red) percentiles calculated specifically within each biome.

We used two different metrics to study the link between either (1) generalized disturbance (human and/or natural) or (2) human pressure (that is landscape modifications) and mycorrhizal strategy of native and non-native plants. The first metric, the SDI, captures the intensity and frequency of both natural and human-caused disturbance events (for example, fires, floods, logging and land conversion), representing a proxy for generalized disturbance. This metric is based on land surface transitions detected across 20 years (1999–2019) of the full Landsat, 5, 7 and 8 surface reflectance collection using the precomputed continuous change detection and classification (CDCC) product^90,91^. Although most of these detected changes probably reflect anthropogenic disturbances, this metric can also capture natural events that produce substantial shifts in vegetation phenology, such as flooding or other ecosystem-level disturbances that alter surface reflectance patterns. From the raw land surface change data provided by the CDCC product^90^, we computed an SDI that accounts for the number and intensity of disturbances over the past 20 years (1999–2019). To do this, we quantified the relative frequency of generalized disturbances at each 30-m pixel within a 250-m grid cell and calculated the Shannon diversity index^92^. Using this approach, a high SDI indicates areas with a high disturbance frequency evenly distributed across space. As SDI is available for the years 1999–2019, we tested the effect of the SDI disturbance on the vegetation plots collected in the same period of time (249,947 plots, see Extended Data Fig. 7 for distribution of SDI values across biomes).

The second metric, the global HMI (v.3)^93^, is a measure of cumulative human pressures on terrestrial ecosystems (excluding Antarctica). HMI includes a wide range of human-associated disturbances, such as built-up areas, croplands, pasture lands, human population density, night-time lights, railways, roads and navigable waterways, capturing both direct land conversion and more diffuse human influence on ecosystems. Where human pressure is high, we expect that disturbance events (fire, ploughing and logging) occur more frequently, but acknowledge that HMI is a very indirect proxy for this. HMI was measured every 5 years between 1990 and 2020 at 300-m resolution and values are in the 0–1.0 range, where 0 indicates no detectable human pressure and 1.0 indicates maximum modelled pressure. We assigned values of HMI to vegetation plots by matching the plot sampling date to the closest previous year of measured HMI, so that the survey would reflect the recent prior disturbance. As HMI values are available between 1990 and 2020, we tested the effect of HMI disturbance only on vegetation plots surveyed between 1991 and 2020 (295,056 plots; see Extended Data Fig. 7 for distribution of HMI values across biomes). The full dataset and R codes are available via Zenodo^94,95^.

### Statistical analyses

Our main objective was to test whether the proportion of non-mycorrhizal and facultative-mycorrhizal species differ between native and non-native subsets of plant species in the vegetation plots and how these differences vary across biomes and under different levels of disturbance and/or human pressure. To investigate these biogeographical patterns in the mycorrhizal strategies of non-native plants, we applied generalized linear mixed-effects models with a binomial error distribution and a logit link function, using the glmmTMB package (v.1.1.11.9000)^96^ in R (v.4.4.0). We also included selected environmental variables as covariates to account for background environmental variation.

#### Global non-mycorrhizal and facultative-mycorrhizal strategies in non-native versus native plants

In our first set of models (global models), we tested whether non-mycorrhizal and facultative-mycorrhizal strategies are more common among non-native plants than native plants globally. That is, is there a higher probability of a non-native plant being non-mycorrhizal or facultative-mycorrhizal relative to native species? Specifically, we first compared non-mycorrhizal species against all other strategies combined to capture the broad contrast between non-mycorrhizal and mycorrhizal strategies (arbuscular-mycorrhizal, ectomycorrhizal and facultative-mycorrhizal) using

$$\begin{array}{l}{\rm{cbind}}\,({\rm{non-mycorrhizal}}\,{\rm{count}},{\rm{arbuscular-mycorrhizal}}\,{\rm{count}}\\+{\rm{ectomycorrhizal}}\,{\rm{count}}+{\rm{facultative-mycorrhizal}}\,{\rm{count}})\end{array}$$

as a response variable. We then broke this down into pairwise comparisons to assess whether patterns differed when each mycorrhizal type was considered separately (non-mycorrhizal versus arbuscular-mycorrhizal, non-mycorrhizal versus ectomycorrhizal and non-mycorrhizal versus facultative-mycorrhizal). This is particularly useful for understanding which mycorrhizal type is over-represented in the non-native flora when the mycorrhizal strategy of non-native plants is mycorrhizal. For analogous facultative-mycorrhizal models, we used the same stepwise approach: first comparing facultative-mycorrhizal against arbuscular-mycorrhizal + non-mycorrhizal to capture the broad contrast and then breaking this down into facultative-mycorrhizal versus arbuscular-mycorrhizal and facultative-mycorrhizal versus non-mycorrhizal. Each model included native status of species (native versus non-native) as a fixed effect and plot identity as a random effect to account for the non-independence of native and non-native proportions within the same plot.

#### Biome variation in non-mycorrhizal and facultative-mycorrhizal strategies

To assess whether differences in mycorrhizal strategies of non-native plant species vary across biomes, we extended the global models by including a two-way interaction between the categorical variable for biome (with ten levels) and native status (native or non-native). The categorical variable biome only comprised ten levels as we filtered out four biomes with a very low count of vegetation plots, namely: tropical coniferous forest biome (28), mangroves (43), tropical deciduous broadleaf forests (115) and flooded grasslands (184). This allowed us to test whether the proportion of non-mycorrhizal or facultative-mycorrhizal in native versus non-native varied with biome.

#### Effect of global disturbance intensity on non-mycorrhizal and facultative-mycorrhizal strategies

To test the influence of disturbance on mycorrhizal type or status proportions at the global scale, we added disturbance as a categorical variable with two levels (low versus high), based on the lowest and highest 25% of the SDI or HMI across all plots globally. Only plots in these two groupings were included in the analyses. Disturbance was included as a two-way interaction term with native status, allowing us to evaluate whether the relative prevalence of non-mycorrhizal and facultative-mycorrhizal species differs between native and non-native groups under contrasting disturbance conditions.

#### Interaction of biome and disturbance on non-mycorrhizal and facultative-mycorrhizal strategies

In a final set of models, we assessed how disturbance interacts with native status and biome by incorporating biome-specific disturbance thresholds. Here low and high disturbance were defined within each biome, using the bottom and top 25% of SDI or HMI values for plots within that biome (Extended Data Fig. 7). This within-biome classification accounts for variation in disturbance intensity observed across regions in our dataset. Importantly, results should only be interpreted quantitatively within each biome, as the disturbance level is specific to each biome. Disturbance level was included as a three-way interaction with native status and biome. To ensure statistical robustness and interpretability, we verified that each biome included a sufficient number of plots representing each combination of disturbance level and biome (minimum plot number 157 using the tundra biome as reference), allowing for meaningful comparisons across interaction terms.

#### Post hoc comparisons and contrasts

We used the emmeans package (v.1.11.1)^97^ to compute estimated marginal means (EMMs) for each factor combination, averaging over other model covariates. From these model-based estimates, we then calculated the difference between groups as:

$${\rm{Difference}}\,{\rm{in}}\,{\rm{means}}={{\rm{EMM}}}_{{\rm{non}}-{\rm{native}}}-{{\rm{EMM}}}_{{\rm{native}}}.$$

A positive difference indicates that, on average, the proportion of non-mycorrhizal or facultative-mycorrhizal species was higher in non-natives than in natives, while a negative difference indicates the opposite (Fig. 4). We applied this procedure at three scales: globally across all plots, within each biome and within each disturbance level nested within each biome. We tested the statistical significance (*P* < 0.05) of these differences using the multcomp package (v.1.4-28)^98^, applying a Benjamini–Hochberg correction for multiple testing. Finally, to assess whether disturbance modified the proportion of non-mycorrhizal and facultative-mycorrhizal within native and non-native groups, we performed manual contrasts using the contrast() function from the emmeans package, applying a difference-of-differences approach. Specifically, we calculated:

$$\begin{array}{rcl}\Delta &=&({{\rm{EMM}}}_{{\rm{non}}-{\rm{native}},{\rm{high}}}-{{\rm{EMM}}}_{{\rm{non}}-{\rm{native}},{\rm{low}}})\\&&-({{\rm{EMM}}}_{{\rm{native}},{\rm{high}}}-{{\rm{EMM}}}_{{\rm{native}},{\rm{low}}}).\end{array}$$

This allowed us to test whether the proportion of non-mycorrhizal and facultative-mycorrhizal changed between low and high disturbance levels. Global maps were visualized using the Equal Earth projection. Spatial data processing, raster manipulations and map generations were performed using the terra R package (v.1.7-83)^99^.

#### Impact of invasion on the native plant mycorrhizal-type composition

The displacement of native species by non-natives can drive non-random shifts in functional community structure, potentially skewing baseline mycorrhizal patterns^100,101^. To assess this impact, we modelled the proportions of mycorrhizal types (non-mycorrhizal versus mycorrhizal) in native species between invaded plots and uninvaded plots using a generalized linear model with a binomial error distribution and a logit link function. The model included an invasion status by biome interaction and environmental variables as covariates. Results showed that the proportion of non-mycorrhizal species in uninvaded plots was generally higher than in invaded plots (Supplementary Table 6). To assess whether this shift in native mycorrhizal types due to invasion would qualitatively alter our main results and conclusions, we adjusted our original native non-mycorrhizal estimates using a biome-specific correction factor derived from the new model described above. This sensitivity analysis (Supplementary Table 7) confirmed that, although invasion alters local mycorrhizal composition, our primary conclusions remain robust to these shifts.

#### Rarefied re-analysis

Within the current dataset, certain regions of the world are considerably undersampled relative to others (Extended Data Fig. 3b). To address this imbalance, we conducted a benchmarking analysis comparing results from the full dataset to those from a resampled dataset that downweighs over-represented regions. Specifically, we rarefied the data by subsampling all biomes to match the number of plots in the montane grassland biome (1,958 plots, biome with the third lowest number of plots) and reran the analysis (global and biome models). Results overall are qualitatively consistent with main presented results (Extended Data Fig. 4b,d; detailed results can be found in Supplementary Tables 3 and 4).

### Reporting summary

Further information on research design is available in the Nature Portfolio Reporting Summary linked to this article.

## Supplementary information

Reporting Summary

Peer Review File

Supplementary Tables Supplementary Tables 1–7 with statistical model outputs.

## Source data

Source Data Statistical source data for Figs. 2, 3 and 4 and Extended Data Figs. 1, 2, 4 and 5.

## Data Availability

The preprocessed dataset is available via Zenodo at https://doi.org/10.5281/zenodo.20123479 (ref. ^94^). Coordinates of the plots have been modified to protect the precise location of the surveyed plots. Source data are provided with this paper.

## Code availability

R codes are available via GitHub at https://github.com/sgc92/Mycorrhizal-strategy-of-non-native-plants-varies-with-biome-and-disturbance/ and via Zenodo at https://doi.org/10.5281/zenodo.17308647 (ref. ^95^).
