# Supplementary material for: Mycorrhizal strategy of non-native plants varies with biome and disturbance
Source: Nat Ecol Evol. 2026 Aug 17;10(9):1644–57. doi: 10.1038/s41559-026-03144-9 (PMC13541620; doi:10.1038/s41559-026-03144-9)
Supplement: Supplementary file 1 [file 41559_2026_3144_MOESM1_ESM.pdf]

nature portfolio

Corresponding author(s): Cazzaniga, Sara Giulia

Last updated by author(s): 2026.05.11

Reporting Summary

Nature Portfolio wishes to improve the reproducibility of the work that we publish. This form provides structure for consistency and transparency in reporting. For further information on Nature Portfolio policies, see our Editorial Policies and the Editorial Policy Checklist.

Statistics

For all statistical analyses, confirm that the following items are present in the figure legend, table legend, main text, or Methods section.

n/a	Confirmed	
☐	☒	The exact sample size (*n*) for each experimental group/condition, given as a discrete number and unit of measurement
☐	☒	A statement on whether measurements were taken from distinct samples or whether the same sample was measured repeatedly
☐	☒	The statistical test(s) used AND whether they are one- or two-sided<br>*Only common tests should be described solely by name; describe more complex techniques in the Methods section.*
☐	☒	A description of all covariates tested
☐	☒	A description of any assumptions or corrections, such as tests of normality and adjustment for multiple comparisons
☐	☒	A full description of the statistical parameters including central tendency (e.g. means) or other basic estimates (e.g. regression coefficient) AND variation (e.g. standard deviation) or associated estimates of uncertainty (e.g. confidence intervals)
☐	☒	For null hypothesis testing, the test statistic (e.g. *F*, *t*, *r*) with confidence intervals, effect sizes, degrees of freedom and *P* value noted<br>*Give P values as exact values whenever suitable.*
☒	☐	For Bayesian analysis, information on the choice of priors and Markov chain Monte Carlo settings
☐	☒	For hierarchical and complex designs, identification of the appropriate level for tests and full reporting of outcomes
☒	☐	Estimates of effect sizes (e.g. Cohen's *d*, Pearson's *r*), indicating how they were calculated

Our web collection on statistics for biologists contains articles on many of the points above.

Software and code

Policy information about availability of computer code

Data collection	no software was used for data collection
Data analysis	R codes have been deposited on Github at https://github.com/sgc92/Mycorrhizal-strategy-of-non-native-plants-varies-with-biome-and-disturbance/ and published in Zenodo at https://doi.org/10.5281/zenodo.17308647 (Cazzaniga & Delavaux, 2026b). On the GitHub page, all R packages used and their their versions are listed (File R version and R packages).<br><br>Statistical packages listed in the manuscript:<br>emmeans v. 1.11.1<br>multcomp v. 1.4-28<br>DHARMa v.0.4.6<br>glmmTMB v.1.1.11.9000<br>terra (v. 1.7-83)<br><br>Cazzaniga, S. G., & Delavaux, C. S. (2026b). Mycorrhizal_strategy_of_non-native_plants_varies_with_biome_and_disturbance (#code). Zenodo. https://doi.org/10.5281/zenodo.20123219

For manuscripts utilizing custom algorithms or software that are central to the research but not yet described in published literature, software must be made available to editors and reviewers. We strongly encourage code deposition in a community repository (e.g. GitHub). See the Nature Portfolio guidelines for submitting code & software for further information.

Data

Policy information about availability of data

All manuscripts must include a data availability statement. This statement should provide the following information, where applicable:

- Accession codes, unique identifiers, or web links for publicly available datasets
- A description of any restrictions on data availability
- For clinical datasets or third party data, please ensure that the statement adheres to our policy

The pre-processed dataset is available at https://doi.org/10.5281/zenodo.20123479 (Cazzaniga & Delavaux, 2026a). Coordinates of the plots have been modified to protect the precise location of the surveyed plots.

Cazzaniga, S. G., & Delavaux, C. S. (2026a). Mycorrhizal_strategy_of_non-native_plants_varies_with_biome_and_disturbance [Dataset]. Zenodo. https://doi.org/10.5281/zenodo.20123479

Research involving human participants, their data, or biological material

Policy information about studies with human participants or human data. See also policy information about sex, gender (identity/presentation), and sexual orientation and race, ethnicity and racism.

Reporting on sex and gender	*Use the terms sex (biological attribute) and gender (shaped by social and cultural circumstances) carefully in order to avoid confusing both terms. Indicate if findings apply to only one sex or gender; describe whether sex and gender were considered in study design; whether sex and/or gender was determined based on self-reporting or assigned and methods used. Provide in the source data disaggregated sex and gender data, where this information has been collected, and if consent has been obtained for sharing of individual-level data; provide overall numbers in this Reporting Summary. Please state if this information has not been collected. Report sex- and gender-based analyses where performed, justify reasons for lack of sex- and gender-based analysis.*
Reporting on race, ethnicity, or other socially relevant groupings	*Please specify the socially constructed or socially relevant categorization variable(s) used in your manuscript and explain why they were used. Please note that such variables should not be used as proxies for other socially constructed/relevant variables (for example, race or ethnicity should not be used as a proxy for socioeconomic status). Provide clear definitions of the relevant terms used, how they were provided (by the participants/respondents, the researchers, or third parties), and the method(s) used to classify people into the different categories (e.g. self-report, census or administrative data, social media data, etc.) Please provide details about how you controlled for confounding variables in your analyses.*
Population characteristics	*Describe the covariate-relevant population characteristics of the human research participants (e.g. age, genotypic information, past and current diagnosis and treatment categories). If you filled out the behavioural & social sciences study design questions and have nothing to add here, write "See above."*
Recruitment	*Describe how participants were recruited. Outline any potential self-selection bias or other biases that may be present and how these are likely to impact results.*
Ethics oversight	*Identify the organization(s) that approved the study protocol.*

Note that full information on the approval of the study protocol must also be provided in the manuscript.

Field-specific reporting

Please select the one below that is the best fit for your research. If you are not sure, read the appropriate sections before making your selection.

☐ Life sciences ☐ Behavioural & social sciences ☒ Ecological, evolutionary & environmental sciences

For a reference copy of the document with all sections, see nature.com/documents/nr-reporting-summary-flat.pdf

Ecological, evolutionary & environmental sciences study design

All studies must disclose on these points even when the disclosure is negative.

Study description	This global observational study combined 440,788 vegetation plots from the sPlot database with plant native status (from GloNAF and POWO) and mycorrhizal strategy (from FungalRoot).The design was factorial, testing interactions between:Native status (native vs. non-native)Biome (14 Olson et al. 2001 biomes; 10 retained in analyses)Disturbance level (low vs. high, based on Structural Disturbance Index – SDI – and Human Modification Index – HMI)Generalized linear mixed models (GLMMs) with plot identity as a random factor tested whether the proportion of non-mycorrhizal (NM) and facultative mycorrhizal (FM) species differed between native and non-native plants across biomes and under different disturbance regimes.
Research sample	Vegetation plot data: from sPlot v4.0, integrating surveys (1888–2022) across all continents.Native status: assigned via GloNAF and POWO; plots with conflicting status were excluded.Mycorrhizal traits: derived from FungalRoot, supplemented at genus level when species data were missing.Environmental and soil variables: extracted from CHELSA (climate) and SoilGrids (soil properties)

	datasets.Disturbance metrics:SDI – computed from 1999–2019 Landsat surface-reflectance change (CDCC).HMI – from Theobald et al. (2025), reflecting cumulative human modification.Biomes were assigned following Olson et al. (2001).
Sampling strategy	The study used pre-existing vegetation plot data compiled in the sPlot 4.0 database, which aggregates >2.5 million standardized vegetation surveys collected worldwide by independent researchers using comparable phytosociological methods.A total of 440,788 plots were selected based on strict inclusion criteria: each had at least 80% of species assigned to a mycorrhizal status and included ≥1 non-native plant species.Sample sizes differ among biomes because data availability varies geographically — some biomes (e.g., temperate forests, grasslands) are extensively surveyed, whereas others (e.g., tundra, tropical coniferous forests) are sparsely sampled. These differences reflect global research and accessibility biases rather than experimental design, but they were statistically accounted for via rarefied re-analysis to ensure robustness to sampling imbalance.
Data collection	Vegetation plot data were originally collected by hundreds of field ecologists and botanists contributing to sPlot, typically following standard Braun-Blanquet or equivalent community survey protocols (species presence or cover recorded per plot).The current study did not perform new field sampling but synthesized existing datasets.Native status was assigned by matching species to GloNAF and POWO databases using geographic coordinates.Mycorrhizal strategy was assigned via FungalRoot, supplemented at genus level.Disturbance indices were derived computationally from Landsat imagery (for SDI) and human modification maps (for HMI), while environmental variables were extracted from CHELSA and SoilGrids rasters.All processing, cleaning, and analysis were conducted in R (v4.4.0) using reproducible scripts available on GitHub and Zenodo ublished in Zenodo at https://doi.org/10.5281/zenodo.17308647 (Cazzaniga & Delavaux, 2026b).<br>Cazzaniga, S. G., & Delavaux, C. S. (2026b). Mycorrhizal_strategy_of_non-native_plants_varies_with_biome_and_disturbance (#code). Zenodo. https://doi.org/10.5281/zenodo.20123219
Timing and spatial scale	Temporal scale: 1899 – 2022 (plots collected over >120 years); disturbance indices aligned with overlapping years (1991–2020 for HMI; 1999–2019 for SDI).Spatial scale: global, covering 14 biomes and >440,000 plots (~1 km² resolution for environmental layers).Analyses were performed globally and per-biome.
Data exclusions	Plots were excluded if:Species had conflicting native status between GloNAF and POWO.<80 % of species in a plot could be assigned a mycorrhizal status.No non-native species were recorded (final dataset included only plots with ≥ 1 non-native species).Biomes with very few plots (<200) were excluded from statistical models.
Reproducibility	This is an observational study, and reproducibility therefore refers to the ability to replicate the analyses rather than the original data collection.All analysis codes and processed datasets are publicly available on Zenodo (respectively DOI: 10.5281/zenodo.17308647 and DOI: 10.5281/zenodo.20123219), enabling full reproduction of data processing and statistical workflows.
Randomization	Randomization was not applicable, as this was an observational study integrating existing vegetation data rather than a manipulative experiment.Instead, sources of non-independence and confounding were controlled statistically:Plot identity was included as a random effect in GLMMs to account for within-plot dependence.Environmental covariates (climate and soil variables) were standardized and included in models to control for background environmental variation.Disturbance variables were categorized (low/high) based on global or biome-specific quantiles to enable structured comparison.
Blinding	Blinding was not used and not applicable because:The analyses relied entirely on pre-existing, publicly available datasets with no subjective assessment of outcomes. All data linking (native status, mycorrhizal assignment, disturbance) was performed computationally using standardized databases and automated scripts, minimizing observer bias.

Did the study involve field work? ☐ Yes ☒ No

Reporting for specific materials, systems and methods

We require information from authors about some types of materials, experimental systems and methods used in many studies. Here, indicate whether each material, system or method listed is relevant to your study. If you are not sure if a list item applies to your research, read the appropriate section before selecting a response.

Materials & experimental systems

n/a	Involved in the study
☒	☐ Antibodies
☒	☐ Eukaryotic cell lines
☒	☐ Palaeontology and archaeology
☒	☐ Animals and other organisms
☒	☐ Clinical data
☒	☐ Dual use research of concern
☒	☐ Plants

Methods

n/a	Involved in the study
☒	☐ ChIP-seq
☒	☐ Flow cytometry
☒	☐ MRI-based neuroimaging

Plants

Seed stocks

Report on the source of all seed stocks or other plant material used. If applicable, state the seed stock centre and catalogue number. If plant specimens were collected from the field, describe the collection location, date and sampling procedures.

Novel plant genotypes

Describe the methods by which all novel plant genotypes were produced. This includes those generated by transgenic approaches, gene editing, chemical/radiation-based mutagenesis and hybridization. For transgenic lines, describe the transformation method, the number of independent lines analyzed and the generation upon which experiments were performed. For gene-edited lines, describe the editor used, the endogenous sequence targeted for editing, the targeting guide RNA sequence (if applicable) and how the editor was applied.

Authentication

Describe any authentication procedures for each seed stock used or novel genotype generated. Describe any experiments used to assess the effect of a mutation and, where applicable, how potential secondary effects (e.g. second site T-DNA insertions, mosiacism, off-target gene editing) were examined.
