# Supplementary material for: Mycorrhizal strategy of non-native plants varies with biome and disturbance
Source: Nat Ecol Evol. 2026 Aug 17;10(9):1644–57. doi: 10.1038/s41559-026-03144-9 (PMC13541620; doi:10.1038/s41559-026-03144-9)
Supplement: Supplementary file 2 [file 41559_2026_3144_MOESM2_ESM.pdf]

nature portfolio

Peer Review File

Mycorrhizal strategy of non-native plants varies with biome and disturbance

Corresponding Author: Dr Sara G Cazzaniga

This file contains all editorial decision letters in order by version, followed by all author rebuttals in order by version.

Version 0:

Reviewer comments:

Reviewer #1

(Remarks to the Author)
Overall this paper is well conducted, and I cannot detect any concerns regarding the statistical analysis. That said, the relative impact of this study is likely limited, being rather confirmatory rather than novel. Ultimately this study is effective at generating hypotheses regarding mechanisms by which NM plants are either over or underrepresented in different biomes, but cannot attempt to address them. Breaking down these contrasts among biomes is effective and presents a nice attempt at identifying mechanisms, but again, is limited in inference.

A strength of this paper is its assessment of whether FM/NM patterns differed when each mycorrhizal type was considered separately (i.e. maximum contrasts).
The authors identification that NM species are dominant in AM dominated landscapes is not necessarily novel, but confirms long-understood patterns.

There are significant limitations to this study, notably the authors do not actually quantify mycorrhizal infection rates. Mycorrhizal infection or colonization rates can vary substantially within a mycorrhizal type, and this should be addressed in the limitations section. That said, because the actual infection rate for putatively mycorrhizal plants can sometimes be close to zero contingent on plant physiology, soil disturbance etc. I think this doesn’t disqualify the paper, but the paper ignores substantial layers of ecology as a result of such a top-down analysis. See Soudzilovskaia et al., 2015 GCB.

One additional question concerns the environmental covariates used in the GLMM. For example, additional rasters are easily available such as high resolution maps of N-dep, which has been repeatedly linked with invasions of grasses in hot/dry biomes. I find this section to be lacking and would like to see an expanded analysis of variables incorporated.

Reviewer #2

(Remarks to the Author)
The manuscript entitled “Mycorrhizal strategy of non-native plants varies with biome and disturbance” by Cazzaniga et al. presents an analysis of a large collection of global vegetation inventories, specifically contrasting mycorrhizal strategies of native versus non-native vegetation. A relationships between mycorrhizal types of plants and their tendency to be succesful invaders has been hypothesized and studied frequently, and therefore this is a prominent issue. Up to date, the issue has not been resolved, especially because of conflicting patterns between different studies/settings. Hence, the fact that the current MS uses a very comprehensive dataset is a clear strength. The findings that the relationship is indeed biome-dependent, and dependent on extent on human impact are therefore a very important step forward.

The MS is very well written, easy to follow, and the analytical approach is sound. As you see below in detailed comments I have very few suggestions for improvement.

To me, one of the most striking surprise is the contrast between the disturbance indices used (SDI and HMI). At nearly each level assessed, they contrast in sign in NM strategies being overrepresented versus Mycorrhizal strategies in non-native plants, at high versus low disturbance. Hence, the overall conclusion that disturbance favors NM types as mentioned in the abstract is contentious. It begs for a further exploration of where and in what way these metrics themselves are themselves positively or negatively correlated (as one would expect a positive correlation?), and hence which aspects of disturbance

may favor NM versus mycorrhizal lifestyles in invasive species. I would understand if such an exploration goes beyond the current MS, but then I would be very careful to draw a general conclusion on the extent to which disturbance modifies the proportion of NM plants in invasive-status.

One other general comment is that invasion intensity is not considered. Given that it's sometimes argued that NM invaders (or those of the opposite mycorrhizal types) can facilitate further invasion (by reducing the positive feedbacks that may exist in the resident community), it is plausible that the success of different mycorrhizal types may depend on the invasion intensity, as well as the distribution of mycorrhizal types in the recipient community.

I suppose these comments indicate that I as a reader see clear merit in the MS, without doubt, and understand that in such a global analysis it is difficult to address many (more detailed) research questions and explorations. Hence, it can serve as an important step forward towards answering these more ultimate questions, and provide impetus in showing there is no general answer to the question whether invaders tend to be one mycorrhizal type over the other.

Specific comments and suggestions
L119: the number of plots reported here contrasts with the number in L136; I think the reason for this disparity maybe versions of the database, or inclusion criteria, but whatever the reason would be good indicate more clearly to avoid confusion.
L325: here it is stated that "most temperate regions are normally dominated by ectomycorrhizal plants" – I think this is an overstatement. One could argue this to be the case for boreal forests perhaps, but temperate regions are very often mixed, especially when not forested.
L330-337: this section is fairly repetitive with redundant statements, best proofread to increase readability.
L405-406: here there is mention of FM species/plants conferring an advantage to non-native establishment. This suggests it is the strategy of the recipient community that provides the advantage, whereas the strategy of the invader is what's meant.
L420: here it is stated that while FM plants tend to be flexible and have broad ranges, they are in fact underrepresented as invasives. On the one hand, there is an implicit bias towards FM designation in more common plants: species can have 0, 1, or more entries in a database as either being colonized by mycorrhizal fungi or not. Only those with sufficient entries can be designated FM (have entries of both being mycorrhized or not), hence there is an increasing chance to be designated FM with increasing entries, as it only takes one count of the opposite type to become FM instead of myc vs NM (mistake or not). On the other hand, common plants may be more likely to have already been naturalized (and so don't count as invasives, but count in the denominator). These considerations don't necessarily negate the conclusion, but are important to keep in mind when drawing ecological conclusions.
L538: yellow should here be green?

Reviewer #3

(Remarks to the Author)
The manuscript entitled "Mycorrhizal strategy of non-native plants varies with biome and disturbance" is based on an impressive global comparison of mycorrhizal strategies between native and non-native floras. The authors also assess the influence of biomes, disturbances and their interaction on native vs. non-native plants mycorrhizal strategies. To address these questions, they use the most up-to-date global databases on vegetation plots (the sPlot database), naturalized non-native plants (Global Naturalized Alien Flora) and native plants distributions (Plants of the World Online), as well as plants mycorrhizal strategies (FungalRoot database). This type of research is fundamental to help us understand the biogeographic patterns of plant invasions. However, I found some issues with the manuscript:

-As I understand (according to lines 190-191) the study is based only on plots where non-native species have become naturalized. In these plots, the presence of non-native plants may have caused a shift in the dominant mycorrhizal strategy of native plants (for example, if some native species have been displaced due to competition with non-native species, and only a particular subset of native species remain in each plot). For instance, in some contexts it is possible that only obligate ectomycorrhizal species are able to compete with non-natives and other mycorrhizal types (such as arbuscular mycorrhizal) have been replaced in invaded plots. As a result, the comparison of mycorrhizal strategies between the non-native flora and the remaining native flora for each plot would not be particularly informative of how advantageous the non-native mycorrhizal strategy was for non-native plant establishment. Therefore, I suggest mycorrhizal strategies for native species should be assessed in plots where non-natives are absent (and also their influence on natives). One option would be to use paired uninvaded plots (100% native) vs invaded plots (non-native > 0).

- Both in the introduction and the discussion sections, the authors fail to describe the main findings of previous relevant literature addressing the same question. In the introduction (lines 73-75) the authors mention that "the role of mycorrhizal strategy in invasion biology remains understudied, and it is unclear which mycorrhizal strategy is associated with non-native plant establishment across different biogeographic contexts", but previous studies have found consistent results showing that facultative mycorrhizal species show higher naturalization success (i.e. facultative mycorrhizal species are more likely to become naturalized, and have become naturalized in more regions or over a larger area than obligate mycorrhizal species and non-mycorrhizal species) (Menzel et al. 2017, Pyzek et al. 2019, Moyano et al. 2020).

In the discussion, the authors state that "facultative mycorrhizal plants do not confer an advantage to non-native establishment" without considering previous studies showing the opposite (Menzel et al. 2017, Pyzek et al. 2019, Moyano et al. 2020). The authors should discuss how their results contrast with previous studies focused on the same question (addressed by different approaches).

Specific comments:

Abstract

Line 39-40: This is only true for one metric of disturbance, while the other metric shows the opposite trend. These contrasting results should be clearly described in the abstract.

Introduction

Line 83-85: I suggest the authors also cite Nuñez et al. (2009) here

Lines 85-89: Menzel et al. (2017) and Moyano et al. (2020) found evidence that facultative mycorrhizal species show higher establishment success outside their native range (i.e. have become established in more regions or a larger area) than obligate mycorrhizal and non-mycorrhizal species. As it is written this sentence ignores their main findings.

Methods

Line 135: Is there a standardized plot size for the sPlot database? If not, what is the range of plot sizes included in this database?

Lines 154-156: Why not consider mycorrhizal types orchid mycorrhizal and ericoid mycorrhizal?

Results and Discussion

Line 379: This sentence contradicts the first sentence of the previous paragraph (lines 362-364), where the authors state "… a trend that was consistent across global, and biome level analyses".
Lines 382-387: This is an interesting point, but it should apply not only to mediterranean forests but also to temperate forests and boreal forests.

Lines 389-390: Again, this sentence contradicts the first sentence of the first paragraph in this subsection (lines 362-364), where the authors describe the consistent effect of disturbance on mycorrhizal strategy

Line 405: The title of this subsection is too strong and definitive. While the authors found that facultative mycorrhizal species are underrepresented when comparing non-native vs native species, other approaches have found evidence that facultative mycorrhizal species show higher naturalization success (i.e. are more likely to become naturalized, have become naturalized in more regions or over a larger area than obligate mycorrhizal species and non-mycorrhizal species) (Menzel et al. 2017, Pysek et al. 2019, Moyano et al. 2020). The authors should discuss how their results contrast with previous relevant literature on the same question (addressed by different approaches).

Lines 454-455: This is only true for one metric of disturbance, while the other shows the opposite trend. These contrasting results should be clearly stated in the conclusion.

Lines 456-460: It is not only an assumption or an idea, there is evidence from previous literature showing that facultative mycorrhizal species are more likely to become naturalized outside their native range and across a wider area (Menzel et al. 2017, Pyzek et al. 2019, Moyano et al. 2020). Further, while the authors provide evidence that facultative mycorrhizal species are underrepresented when comparing non-native vs. native flora, this does not necessarily refute previous findings addressing this same question using different approaches.

References

Menzel A, Hempel S, Klotz S, et al (2017) Mycorrhizal status helps explain invasion success of alien plant species. Ecology 98:92–102. https://doi.org/10.1002/ecy.1621

Moyano J, Dickie IA, Rodriguez-Cabal MA, Nuñez MA (2020) Patterns of plant naturalization show that facultative mycorrhizal plants are more likely to succeed outside their native Eurasian ranges. Ecography 43:648–659. https://doi.org/10.1111/ecog.04877

Nuñez MA, Horton TR, Simberloff D (2009) Lack of belowground mutualisms hinders Pinaceae invasions. Ecology 90:2352–2359. https://doi.org/10.1890/08-2139.1

Pyšek P, Guo W-Y, Štajerová K, et al (2019) Facultative mycorrhizal associations promote plant naturalization worldwide. Ecosphere 10:e02937. https://doi.org/10.1002/ecs2.2937

Decision Letter:

21st November 2025

*Please ensure you delete the link to your author homepage in this e-mail if you wish to forward it to your co-authors.

Dear Sara,

Thank you for your patience while your manuscript entitled "Mycorrhizal strategy of non-native plants varies with biome and disturbance" was under review. As you will see from the reports below, the reviewers find your work of interest but raise a number of concerns that will need to be addressed with additional analyses and textual changes. Therefore, we would like to invite a revised manuscript along with a point-by-point response to the reviewers.

We are committed to providing a fair and constructive peer-review process. Please do not hesitate to contact me if you have any questions or there are specific requests from the reviewers that you believe are technically impossible or unlikely to yield a meaningful outcome.

When revising your manuscript:

* Please provide the manuscript in Microsoft Word format and highlight all changes in the same file or a copy.

* Include a “Response to reviewers” document detailing, point-by-point, how you addressed each reviewer comment. If no action was taken to address a point, you must provide a compelling argument. This response will be sent back to the reviewers along with the revised manuscript.

* If you have not done so already, please begin to revise your manuscript so that it conforms to our Article format instructions at http://www.nature.com/natecolevol/info/final-submission. Refer also to any guidelines provided in this letter.

* Include a revised version of the reporting checklist.

* Extended Data Figures - please ensure that any supplementary figures and tables that are crucial to the manuscript’s conclusions are converted into Extended Data figures and tables to increase visibility of these data. Extended Data figures and tables are online-only (present in the online PDF and full-text HTML versions of the paper), peer-reviewed display items that provide essential background to the article but are not included in the main article due to space constraints. A maximum of ten Extended Data display items (figures and tables) is permitted.

We hope to receive your revised manuscript within three months. If you cannot send it within this time, please let us know. We will be happy to consider your revision so long as nothing similar has been accepted for publication at Nature Ecology & Evolution or published elsewhere. Once ready, olease use the link below to submit your revised manuscript and related files:

Link Redacted

Note: This URL links to your confidential home page and associated information about manuscripts you may have submitted, or that you are reviewing for us. If you wish to forward this email to co-authors, please delete the link to your homepage.

Nature Ecology & Evolution is committed to improving transparency in authorship. As part of our efforts in this direction, we are now requesting that all authors identified as ‘corresponding author’ on published papers create and link their Open Researcher and Contributor Identifier (ORCID) with their account on the Manuscript Tracking System (MTS), prior to acceptance. ORCID helps the scientific community achieve unambiguous attribution of all scholarly contributions. You can create and link your ORCID from the home page of the MTS by clicking on ‘Modify my Springer Nature account’. For more information please visit please visit www.springernature.com/orcid.

We look forward to seeing the revised manuscript.

[redacted]

****

Reviewers' comments:

Reviewer #1 (Remarks to the Author):

Overall this paper is well conducted, and I cannot detect any concerns regarding the statistical analysis. That said, the relative impact of this study is likely limited, being rather confirmatory rather than novel. Ultimately this study is effective at generating hypotheses regarding mechanisms by which NM plants are either over or underrepresented in different biomes, but cannot attempt to address them. Breaking down these contrasts among biomes is effective and presents a nice attempt at identifying mechanisms, but again, is limited in inference.

A strength of this paper is its assessment of whether FM/NM patterns differed when each mycorrhizal type was considered separately (i.e. maximum contrasts).
The authors identification that NM species are dominant in AM dominated landscapes is not necessarily novel, but confirms long-understood patterns.

There are significant limitations to this study, notably the authors do not actually quantify mycorrhizal infection rates. Mycorrhizal infection or colonization rates can vary substantially within a mycorrhizal type, and this should be addressed in the limitations section. That said, because the actual infection rate for putatively mycorrhizal plants can sometimes be close to zero contingent on plant physiology, soil disturbance etc. I think this doesn't disqualify the paper, but the paper ignores substantial layers of ecology as a result of such a top-down analysis. See Soudzilovskaia et al., 2015 GCB.

One additional question concerns the environmental covariates used in the GLMM. For example, additional rasters are easily available such as high resolution maps of N-dep, which has been repeatedly linked with invasions of grasses in hot/dry biomes. I find this section to be lacking and would like to see an expanded analysis of variables incorporated.

Reviewer #2 (Remarks to the Author):

The manuscript entitled “Mycorrhizal strategy of non-native plants varies with biome and disturbance” by Cazzaniga et al. presents an analysis of a large collection of global vegetation inventories, specifically contrasting mycorrhizal strategies of native versus non-native vegetation. A relationships between mycorrhizal types of plants and their tendency to be succesful invaders has been hypothesized and studied frequently, and therefore this is a prominent issue. Up to date, the issue has not been resolved, especially because of conflicting patterns between different studies/settings. Hence, the fact that the current MS uses a very comprehensive dataset is a clear strength. The findings that the relationship is indeed biome-dependent, and dependent on extent on human impact are therefore a very important step forward.

The MS is very well written, easy to follow, and the analytical approach is sound. As you see below in detailed comments I have very few suggestions for improvement.

To me, one of the most striking surprise is the contrast between the disturbance indices used (SDI and HMI). At nearly each level assessed, they contrast in sign in NM strategies being overrepresented versus Mycorrhizal strategies in non-native plants, at high versus low disturbance. Hence, the overall conclusion that disturbance favors NM types as mentioned in the abstract is contentious. It begs for a further exploration of where and in what way these metrics themselves are themselves positively or negatively correlated (as one would expect a positive correlation?), and hence which aspects of disturbance may favor NM versus mycorrhizal lifestyles in invasive species. I would understand if such an exploration goes beyond the current MS, but then I would be very careful to draw a general conclusion on the extent to which disturbance modifies the proportion of NM plants in invasive-status.

One other general comment is that invasion intensity is not considered. Given that it's sometimes argued that NM invaders (or those of the opposite mycorrhizal types) can facilitate further invasion (by reducing the positive feedbacks that may exist in the resident community), it is plausible that the success of different mycorrhizal types may depend on the invasion intensity, as well as the distribution of mycorrhizal types in the recipient community.

I suppose these comments indicate that I as a reader see clear merit in the MS, without doubt, and understand that in such a global analysis it is difficult to address many (more detailed) research questions and explorations. Hence, it can serve as an important step forward towards answering these more ultimate questions, and provide impetus in showing there is no general answer to the question whether invaders tend to be one mycorrhizal type over the other.

Specific comments and suggestions
L119: the number of plots reported here contrasts with the number in L136; I think the reason for this disparity maybe versions of the database, or inclusion criteria, but whatever the reason would be good indicate more clearly to avoid confusion.
L325: here it is stated that “most temperate regions are normally dominated by ectomycorrhizal plants” – I think this is an overstatement. One could argue this to be the case for boreal forests perhaps, but temperate regions are very often mixed, especially when not forested.
L330-337: this section is fairly repetitive with redundant statements, best proofread to increase readability.
L405-406: here there is mention of FM species/plants conferring an advantage to non-native establishment. This suggests it is the strategy of the recipient community that provides the advantage, whereas the strategy of the invader is what's meant.
L420: here it is stated that while FM plants tend to be flexible and have broad ranges, they are in fact underrepresented as invasives. On the one hand, there is an implicit bias towards FM designation in more common plants: species can have 0, 1, or more entries in a database as either being colonized by mycorrhizal fungi or not. Only those with sufficient entries can be designated FM (have entries of both being mycorrhized or not), hence there is an increasing chance to be designated FM with increasing entries, as it only takes one count of the opposite type to become FM instead of myc vs NM (mistake or not). On the other hand, common plants may be more likely to have already been naturalized (and so don't count as invasives, but count in the denominator). These considerations don't necessarily negate the conclusion, but are important to keep in mind when drawing ecological conclusions.
L538: yellow should here be green?

Reviewer #3 (Remarks to the Author):

The manuscript entitled “Mycorrhizal strategy of non-native plants varies with biome and disturbance” is based on an impressive global comparison of mycorrhizal strategies between native and non-native floras. The authors also assess the influence of biomes, disturbances and their interaction on native vs. non-native plants mycorrhizal strategies. To address these questions, they use the most up-to-date global databases on vegetation plots (the sPlot database), naturalized non-native plants (Global Naturalized Alien Flora) and native plants distributions (Plants of the World Online), as well as plants mycorrhizal strategies (FungalRoot database). This type of research is fundamental to help us understand the biogeographic patterns of plant invasions. However, I found some issues with the manuscript:

-As I understand (according to lines 190-191) the study is based only on plots where non-native species have become naturalized. In these plots, the presence of non-native plants may have caused a shift in the dominant mycorrhizal strategy of native plants (for example, if some native species have been displaced due to competition with non-native species, and only a particular subset of native species remain in each plot). For instance, in some contexts it is possible that only obligate ectomycorrhizal species are able to compete with non-natives and other mycorrhizal types (such as arbuscular mycorrhizal) have been replaced in invaded plots. As a result, the comparison of mycorrhizal strategies between the non-native flora and the remaining native flora for each plot would not be particularly informative of how advantageous the non-native mycorrhizal strategy was for non-native plant establishment. Therefore, I suggest mycorrhizal strategies for native species should be assessed in plots where non-natives are absent (and also their influence on natives). One option would be to use paired uninvaded plots (100% native) vs invaded plots (non-native > 0).

- Both in the introduction and the discussion sections, the authors fail to describe the main findings of previous relevant literature addressing the same question. In the introduction (lines 73-75) the authors mention that “the role of mycorrhizal strategy in invasion biology remains understudied, and it is unclear which mycorrhizal strategy is associated with non-native plant establishment across different biogeographic contexts”, but previous studies have found consistent results showing that facultative mycorrhizal species show higher naturalization success (i.e. facultative mycorrhizal species are more likely to become naturalized, and have become naturalized in more regions or over a larger area than obligate mycorrhizal species and non-mycorrhizal species) (Menzel et al. 2017, Pyzek et al. 2019, Moyano et al. 2020).

In the discussion, the authors state that “facultative mycorrhizal plants do not confer an advantage to non-native establishment” without considering previous studies showing the opposite (Menzel et al. 2017, Pyzek et al. 2019, Moyano et al. 2020). The authors should discuss how their results contrast with previous studies focused on the same question (addressed by different approaches).

Specific comments:

Abstract

Line 39-40: This is only true for one metric of disturbance, while the other metric shows the opposite trend. These contrasting results should be clearly described in the abstract.

Introduction

Line 83-85: I suggest the authors also cite Nuñez et al. (2009) here

Lines 85-89: Menzel et al. (2017) and Moyano et al. (2020) found evidence that facultative mycorrhizal species show higher establishment success outside their native range (i.e. have become established in more regions or a larger area) than obligate mycorrhizal and non-mycorrhizal species. As it is written this sentence ignores their main findings.

Methods

Line 135: Is there a standardized plot size for the sPlot database? If not, what is the range of plot sizes included in this database?

Lines 154-156: Why not consider mycorrhizal types orchid mycorrhizal and ericoid mycorrhizal?

Results and Discussion

Line 379: This sentence contradicts the first sentence of the previous paragraph (lines 362-364), where the authors state “… a trend that was consistent across global, and biome level analyses”.
Lines 382-387: This is an interesting point, but it should apply not only to mediterranean forests but also to temperate forests and boreal forests.

Lines 389-390: Again, this sentence contradicts the first sentence of the first paragraph in this subsection (lines 362-364), where the authors describe the consistent effect of disturbance on mycorrhizal strategy

Line 405: The title of this subsection is too strong and definitive. While the authors found that facultative mycorrhizal species are underrepresented when comparing non-native vs native species, other approaches have found evidence that facultative mycorrhizal species show higher naturalization success (i.e. are more likely to become naturalized, have become naturalized in more regions or over a larger area than obligate mycorrhizal species and non-mycorrhizal species) (Menzel et al. 2017, Pysek et al. 2019, Moyano et al. 2020). The authors should discuss how their results contrast with previous relevant literature on the same question (addressed by different approaches).

Lines 454-455: This is only true for one metric of disturbance, while the other shows the opposite trend. These contrasting results should be clearly stated in the conclusion.

Lines 456-460: It is not only an assumption or an idea, there is evidence from previous literature showing that facultative mycorrhizal species are more likely to become naturalized outside their native range and across a wider area (Menzel et al. 2017, Pyzek et al. 2019, Moyano et al. 2020). Further, while the authors provide evidence that facultative mycorrhizal species are underrepresented when comparing non-native vs. native flora, this does not necessarily refute previous findings addressing this same question using different approaches.

References

Menzel A, Hempel S, Klotz S, et al (2017) Mycorrhizal status helps explain invasion success of alien plant species. Ecology 98:92–102. https://doi.org/10.1002/ecy.1621

Moyano J, Dickie IA, Rodriguez-Cabal MA, Nuñez MA (2020) Patterns of plant naturalization show that facultative mycorrhizal plants are more likely to succeed outside their native Eurasian ranges. Ecography 43:648–659. https://doi.org/10.1111/ecog.04877

Nuñez MA, Horton TR, Simberloff D (2009) Lack of belowground mutualisms hinders Pinaceae invasions. Ecology 90:2352–2359. https://doi.org/10.1890/08-2139.1

Pyšek P, Guo W-Y, Štajerová K, et al (2019) Facultative mycorrhizal associations promote plant naturalization worldwide. Ecosphere 10:e02937. https://doi.org/10.1002/ecs2.2937

Version 1:

Reviewer comments:

Reviewer #1

(Remarks to the Author)
I appreciate inclusion of disturbance metrics, and for including N-P dep rasters to probe their role in influencing invasion trajectories. I am satisfied with this inclusion.

Regarding the novelty of this study, the causative inference of this study is necessarily limited. This is inherent to all such studies using plot level data like this. The introduction still claims to be testing hypotheses. I believe this is misleading, given the inherent correlative nature of this study. I applaud the authors for their discussion of these limitations in the Discussion section, but the Introduction should be tempered to better reflect the actual study.

I appreciate the inclusion of a sentence on mycorrhizal colonization in the limitations section, but more in depth description as to how to differences in mycorrhizal reliance could influence these patterns would be helpful.

Reviewer #2

(Remarks to the Author)
I have carefully read the rebuttal and the revised MS of "Mycorrhizal strategy of non-native plants varies with biome and disturbance" by Cazzaniga et al. and am pleased with the improvements made by the authors. I believe that they have sufficiently and carefully addressed concerns raised by me and the other reviewers. Congratulations to those authors that have carried out the revisions, additional analyses, and drafting of rebuttal.

Reviewer #3

(Remarks to the Author)
The authors have addressed all my comments and suggestions. I particularly appreciate that they carried out a validation analysis to address the issue I described in my first comment (that the establishment of non-native species may shift the

resident community's mycorrhizal profile).

Well done and thank you for this valuable contribution to our current understanding of biogeographic patterns of plant invasions.

I have no further comments

Decision Letter:

20th March 2026

Dear Sara,

Your revised manuscript "Mycorrhizal strategy of non-native plants varies with biome and disturbance" (NATECOLEVOL-25103503A) has now been seen by the original reviewers, whose comments you will find below. Based on the feedback received, we will be happy in principle to publish it in Nature Ecology & Evolution, pending minor revisions to satisfy the reviewers' final requests and to comply with our editorial and formatting guidelines.

Please provide by email an updated Reporting Summary. The form is available here: https://www.nature.com/documents/nr-reporting-summary.pdf

Once we receive the Reporting Summary, we will perform detailed checks on your paper and send you a checklist detailing our editorial and formatting requirements in about a week. Please do not upload the final materials and make any revisions until you receive this additional information from us.

Thank you again for your interest in Nature Ecology & Evolution. Please do not hesitate to contact me if you have any questions.

Best wishes,

[redacted]

PS Lest I forget to mention it in the checklist, please note that we perplexed by Reviewer 1's apparent suggestion that a correlative study cannot test hypotheses but we agree that the wording in the last paragraph of the Introduction would better match an experimental paper. Please include a sentence or two acknowledging the caveats of testing causal relationships and mechanisms from the kind of data used here.

****

Reviewer #1 (Remarks to the Author):

I appreciate inclusion of disturbance metrics, and for including N-P dep rasters to probe their role in influencing invasion trajectories. I am satisfied with this inclusion.

Regarding the novelty of this study, the causative inference of this study is necessarily limited. This is inherent to all such studies using plot level data like this. The introduction still claims to be testing hypotheses. I believe this is misleading, given the inherent correlative nature of this study. I applaud the authors for their discussion of these limitations in the Discussion section, but the Introduction should be tempered to better reflect the actual study.

I appreciate the inclusion of a sentence on mycorrhizal colonization in the limitations section, but more in depth description as to how to differences in mycorrhizal reliance could influence these patterns would be helpful.

Reviewer #2 (Remarks to the Author):

I have carefully read the rebuttal and the revised MS of "Mycorrhizal strategy of non-native plants varies with biome and disturbance" by Cazzaniga et al. and am pleased with the improvements made by the authors. I believe that they have sufficiently and carefully addressed concerns raised by me and the other reviewers. Congratulations to those authors that have carried out the revisions, additional analyses, and drafting of rebuttal.

Reviewer #3 (Remarks to the Author):

The authors have addressed all my comments and suggestions. I particularly appreciate that they carried out a validation analysis to address the issue I described in my first comment (that the establishment of non-native species may shift the resident community's mycorrhizal profile).

Well done and thank you for this valuable contribution to our current understanding of biogeographic patterns of plant invasions.

I have no further comments

Open Access This Peer Review File is licensed under a Creative Commons Attribution 4.0 International License, which permits use, sharing, adaptation, distribution and reproduction in any medium or format, as long as you give appropriate credit to the original author(s) and the source, provide a link to the Creative Commons license, and indicate if changes were made.
In cases where reviewers are anonymous, credit should be given to 'Anonymous Referee' and the source.
The images or other third party material in this Peer Review File are included in the article's Creative Commons license, unless indicated otherwise in a credit line to the material. If material is not included in the article's Creative Commons license and your intended use is not permitted by statutory regulation or exceeds the permitted use, you will need to obtain permission directly from the copyright holder.
To view a copy of this license, visit https://creativecommons.org/licenses/by/4.0/

Reviewer 1

Overall this paper is well conducted, and I cannot detect any concerns regarding the statistical analysis. That said, the relative impact of this study is likely limited, being rather confirmatory rather than novel. Ultimately this study is effective at generating hypotheses regarding mechanisms by which NM plants are either over or underrepresented in different biomes, but cannot attempt to address them. Breaking down these contrasts among biomes is effective and presents a nice attempt at identifying mechanisms, but again, is limited in inference.

We thank the reviewer for this thoughtful comment. We agree that, in the previous version of the manuscript, the novelty of our study may not have been sufficiently clear. In response to this concern, we have substantially revised the manuscript to more explicitly highlight how our work moves beyond confirmatory evidence.

Specifically, our study is novel in (i) providing the first global-scale empirical test of hypotheses on mycorrhizal association and type across biomes, rather than relying on localized or regional patterns; (ii) explicitly contrasting different mycorrhizal types at the biome level to assess context dependency; and (iii) integrating disturbance metrics as a key, yet rarely considered, mediator of invasion patterns. While some elements of these patterns have been suggested in smaller-scale studies, their joint evaluation at a global and biome level represents a critical advance for macroecological inference.

We have revised the Introduction accordingly to ensure that these contributions are clearly communicated to the reader, and we have added a sentence to highlight how we address the current gaps.

Introduction [L121-125]:

> *"To address these inconsistencies, our study evaluates mycorrhizal strategies on a global scale that accounts for both environmental variation and disturbance, offering a broader empirical basis for understanding the role of mycorrhizal associations in non-native plant establishment."*

A strength of this paper is its assessment of whether FM/NM patterns differed when each mycorrhizal type was considered separately (i.e. maximum contrasts). The authors identification that NM species are dominant in AM dominated landscapes is not necessarily novel, but confirms long-understood patterns.

We thank the reviewer for highlighting the strength of our approach and we agree that the prevalence of NM species in AM landscapes has been discussed in ecological theory. However, the novelty of our work lies in demonstrating that this pattern is not static, but varies significantly when considering the native status of plants and the specific type of disturbance on a global scale.

Specifically, we show that non-native NM species are significantly more frequent in grassland and desert biomes than their native counterparts, suggesting a specific invasion advantage in these contexts. Furthermore, we show that disturbance acts as a critical filter that can override or modify long-understood patterns: Higher levels of generalized disturbance predict an increase in the NM strategy among non-native species, even in systems not dominated by AM fungi; Conversely, human-driven disturbance favors mycorrhizal non-natives regardless of the dominant mycorrhizal type in the landscape. Our study identifies disturbance type as a filter that favors different mycorrhizal strategies, which provides novel understanding of non-native plant establishment than previously available.

There are significant limitations to this study, notably the authors do not actually quantify mycorrhizal infection rates. Mycorrhizal infection or colonization rates can vary substantially within a mycorrhizal type, and this should be addressed in the limitations section. That said, because the actual infection rate for putatively mycorrhizal plants can sometimes be close to zero contingent on plant physiology, soil disturbance etc. I think this doesn't disqualify the paper, but the paper ignores substantial layers of ecology as a result of such a top-down analysis. See Soudzilovskaia et al., 2015 GCB.

We acknowledge that treating mycorrhizal status as a binary or categorical trait is a relatively coarse approach. We further agree that mycorrhizal colonization could be an important consideration to include in such a study. Root colonization intensity can vary widely within a given mycorrhizal type and across environmental contexts, and may not be a consistent proxy for functional outcomes such as host benefit (i.e. mycorrhizal growth response). However, in a global analysis such as ours, quantifying colonization and/or growth response for thousands of species across all sites is unfortunately not possible due to limited and uneven taxonomic and

geographic coverage of available data. Therefore, we suggest that future work would benefit greatly by incorporating species level colonization and/or growth-response information (mycorrhizal benefit), building off existing such databases (e.g. mycoDB), where these data are available and comparable. We have now added text to the Limitations section [Lines 491-505] where we discuss how additional information such as mycorrhizal colonization and growth benefit could build on our assessment:

> *"Finally, although we used the most up-to-date empirical data from the FungalRoot database, associated mycorrhizal information remains incomplete, covering only a small fraction of the global flora (Meng et al., 2023), with marked geographic and taxonomic biases. In addition, where species-level information was unavailable, mycorrhizal status and type were inferred from genus-level assignments, an approach that enables global coverage but introduces uncertainty due to documented within-genus variability in mycorrhizal associations (Bueno et al., 2019; Meng et al., 2023). Further, these assignments may reflect both genuine intra-specific variability and limitations in sampling intensity or diagnostic resolution (e.g. for FM), which can introduce uncertainty in mycorrhizal classification. Beyond taxonomic coverage, an ecological limitation of our analysis is the use of categorical mycorrhizal assignments rather than quantitative rates of colonization or resulting fitness outcomes (i.e. mycorrhizal growth response). This underscores the need for more species- and population-level assessments of mycorrhizal type, colonization rates (Soudzilovskaia et al., 2015), and growth benefit (Chaudhary et al., 2016)."*

One additional question concerns the environmental covariates used in the GLMM. For example, additional rasters are easily available such as high resolution maps of N-dep, which has been repeatedly linked with invasions of grasses in hot/dry biomes. I find this section to be lacking and would like to see an expanded analysis of variables incorporated.

We agree that nitrogen (N) and phosphorus (P) are key drivers of plant nutritional mutualisms. Following the Reviewer's suggestion, we have now performed an additional GLMM analysis incorporating global nitrogen concentrations (SoilGrids; Hengl et al., 2017) and topsoil Olsen phosphorus concentrations (McDowell et al., 2023).

We initially omitted these rasters because global nutrient maps often rely on sparse ground-truth data in many regions, leading to high interpolation errors that may not capture the fine-scale local heterogeneity dictating plant-level nutrient availability. Our sensitivity analysis confirms that the inclusion of N and P as covariates did not qualitatively alter our findings; the calculated differences between native and non-native proportions of mycorrhizal types remained consistent across almost all biomes.

One minor exception was observed in Xeric deserts and shrublands, where the inclusion of nutrients predicted no difference in strategy, whereas our main model predicted an NM preference for non-natives. However, direct comparisons are difficult as the sample size for the nutrient-inclusive model was significantly lower (387,593 retained plots) than our original dataset. Consequently, we have included these results in the Supplementary Materials (Table S5, Figure S7) and added a statement in the Methods [Lines 207-214] noting that our primary conclusions remain robust even when accounting for soil nutrient gradients.

> *Methods [L207]: "To evaluate the influence of soil nutrients, we conducted a sensitivity analysis incorporating soil nitrogen (Hengl et al., 2017) and phosphorus (McDowell et al., 2023). Due to high spatial uncertainty in global nutrient rasters, these were analyzed in separate models to avoid over-interpreting their causal weight. Results remained qualitatively consistent with our primary findings, demonstrating that observed patterns are robust to the inclusion of soil nutrient gradients (Supplementary Table S5, Supplementary Figure S7), with minor variations in Xeric deserts likely due to reduced sample sizes (387,593 plots)."*

<u>Reviewer 2</u>

The manuscript entitled "Mycorrhizal strategy of non-native plants varies with biome and disturbance" by Cazzaniga et al. presents an analysis of a large collection of global vegetation inventories, specifically contrasting mycorrhizal strategies of native versus non-native vegetation. A relationships between mycorrhizal types of plants and their tendency to be succesful invaders has been hypothesized and studied frequently, and therefore this is a prominent issue. Up to date, the issue has not been resolved, especially because of conflicting patterns between different studies/settings. Hence, the fact that the current MS uses a very comprehensive

dataset is a clear strength. The findings that the relationship is indeed biome-dependent, and dependent on extent on human impact are therefore a very important step forward.

The MS is very well written, easy to follow, and the analytical approach is sound. As you see below in detailed comments I have very few suggestions for improvement.

We thank the Reviewer for the positive evaluation of our manuscript and constructive comments to improve our work. We agree that our work contributes meaningfully to addressing competing hypotheses of the role of mycorrhizal associations in invasion patterns and hope that it will be a starting point for future regional and experimental work. Please find detailed responses to comments below.

To me, one of the most striking surprise is the contrast between the disturbance indices used (SDI and HMI). At nearly each level assessed, they contrast in sign in NM strategies being overrepresented versus Mycorrhizal strategies in non-native plants, at high versus low disturbance. Hence, the overall conclusion that disturbance favors NM types as mentioned in the abstract is contentious. It begs for a further exploration of where and in what way these metrics themselves are themselves positively or negatively correlated (as one would expect a positive correlation?), and hence which aspects of disturbance may favor NM versus mycorrhizal lifestyles in invasive species. I would understand if such an exploration goes beyond the current MS, but then I would be very careful to draw a general conclusion on the extent to which disturbance modifies the proportion of NM plants in invasive-status.

We agree that a broad generalization that disturbance favors NM types is not warranted given the divergent patterns observed between our two metrics. It is indeed interesting that these two metrics influence proportions of mycorrhizal types in different ways. Rather than indicating a single, uniform effect of disturbance, our results suggest that different dimensions of disturbance generate contrasting selective environments for mycorrhizal strategies, highlighting testable hypotheses about how fungal persistence, propagule pressure, and host–symbiont dependence mediate invasion success. These indices reflect different disturbance pressures on plant-fungal associations. SDI (generalized disturbance) reflects the frequency and intensity of episodic events (e.g., fire, grazing, flooding) that likely mechanically disrupt soil fungal structures. This likely degrades the mycorrhizal community and reduces mycorrhizal propagule pressure (Hogberg & Piearce, 1986; Sheldrake et al., 2017), favoring the NM strategy in non-native plants. In contrast, HMI (human disturbance) reflects stable anthropogenic infrastructure and population density. In these systems, mycorrhizal fungal communities may persist or be actively spread by human activity (e.g. via overwhelmingly mycorrhizal crops and ornamental species), potentially favoring mycorrhizal non-natives. We tested the correlation between the SDI and HMI and found that while the relationship is statistically significant ($p < 0.001$) due to the high power of our dataset, the correlation is negligible (Pearson's $r = -0.03$). This confirms that these metrics capture fundamentally distinct ecological and anthropogenic dimensions.

Following the Reviewer's suggestion, we have carefully updated the manuscript to ensure the difference in metrics and associated interpretation of results is reflected throughout:

> *L 39-42 "Disturbance type and intensity emerged as key drivers of mycorrhizal strategy of non-native species. Higher generalized disturbance predicted non-native species to be more non-mycorrhizal, while higher human disturbance predicted non-native species to be more mycorrhizal.*
>
> *Section Titles [Line 404]: "Mycorrhizal strategy of non-native plants depends on disturbance intensity and type"*
>
> *Results*
> *On the effect of SDI [Lines 405-429]: "We found that generalized disturbance (SDI) increases the probability of non-native species being non-mycorrhizal (NM) compared to natives at the global level and in the majority of biomes (Figure 3 A&C). This pattern was especially pronounced in tropical grasslands and shrublands, deserts and xeric shrublands, and tropical moist broadleaf forests. These biomes are typically dominated by arbuscular mycorrhizal (AM) associations (Read, 1991), yet are increasingly subject to natural (e.g., fire, grazing) and anthropogenic (e.g., land conversion, deforestation) disturbances (Morris, 2010; Saygın et al., 2011; van Etten et al., 2022; Whitmore & Burslem, 1996). Since AM fungi have limited dispersal capacity (Kivlin et al., 2011), such disturbances can degrade AM fungal inoculum, reducing establishment opportunities for AM-dependent species. Consequently, NM species – often ruderal, disturbance-adapted, and unconstrained by symbiont dependence – can rapidly exploit these conditions (Brundrett, 1991; Peat & Fitter, 1993). This helps*

explain the consistent rise of NM strategies among non-natives in highly disturbed AM systems (Reinhart & Callaway, 2006; Richardson et al., 2000; Vogelsang & Bever, 2009). In contrast, the opposite pattern was found in Mediterranean forests: increased disturbance (SDI) was associated with higher proportions of mycorrhizal species among non-natives (Figure 3A&C). Unlike AM-dominated systems, these forests often host ectomycorrhizal (EM) or mixed AM-EM long-lived woody perennials (Gerz et al., 2019; Read, 1991). These systems may thus retain functional mycorrhizal associations even under disturbance due to the resilience of trees and associated EM fungi, which produce resistant propagules, disperse more effectively, and can persist independently of hosts (Horton & Horton, 2017; Policelli et al., 2022; Smith & Read, 2008). Specifically, the frequent fire and drought of Mediterranean ecosystems may have selected for EM spore banks that are more resilient than those in boreal or temperate forests, where disturbance leads to severe fungal biomass decline (Buscardo et al., 2010; Dahlberg, 2002; Livne-Luzon et al., 2021)."

On the effect of HMI [Lines 431-447]: "The Human Modification Index (HMI), reflecting more stable human pressures such as urbanization and infrastructure, was generally associated with increased prevalence of mycorrhizal species among non-natives (Figure 3 B&D; Supplementary Figure S4 A&B). These results align with results by Clavel et al., (2024), who showed that roadside disturbance promoted mycorrhizal species. Specifically, increasing human disturbance was associated with a higher proportion of mycorrhizal non-natives in grasslands, tropical moist broadleaf forests, boreal forests, and tundra, biomes that were more likely to be non-mycorrhizal under high SDI. In contrast, for Mediterranean and temperate forest biomes (both coniferous and broadleaf), increased human disturbance was associated with higher proportions of non-mycorrhizal non-natives. These contrasting patterns between SDI and HMI underscore the need to consider multiple disturbance types when assessing invasion dynamics. Independence from mycorrhizal fungi may confer an advantage in systems with degraded fungal communities or altered nutrient regimes, where NM species can bypass the carbon costs of symbiosis (Hogberg & Piearce, 1986; Sheldrake et al., 2017; Yu et al., 2022). In contrast, associations with mycorrhizal fungi may confer an advantage to non-native species in less frequently disturbed, human-modified systems where fungal partners persist or are actively spread through human activities (e.g. mycorrhizal crops and ornamental species)."

We emphasize that these interpretations reflect broad-scale associations rather than direct causal mechanisms, and are intended to motivate targeted regional, experimental, and trait-based studies that can explicitly test these processes.

Conclusions [Lines 515-521]: "Moreover, our results show that while disturbance is a key predictor of mycorrhizal strategy, the direction of this effect hinges on the nature of the disturbance. Specifically, generalized disturbance increases the proportion of non-mycorrhizal, whereas human disturbance favors mycorrhizal non-native species. These results suggest distinct underlying mechanisms: while sustained and frequent disturbance likely depletes fungal inoculum, stable anthropogenic pressures appear to preserve, or even facilitate, mutualisms between fungi and non-native plant species."

One other general comment is that invasion intensity is not considered. Given that it's sometimes argued that NM invaders (or those of the opposite mycorrhizal types) can facilitate further invasion (by reducing the positive feedbacks that may exist in the resident community), it is plausible that the success of different mycorrhizal types may depend on the invasion intensity, as well as the distribution of mycorrhizal types in the recipient community.

We agree that invasion intensity is an important factor to consider. While a full assessment of invasion intensity to pinpoint trends of mycorrhizal 'invasion meltdown' would ideally require long-term temporal data (which is currently unavailable using our global dataset), we have incorporated a proxy for invasion intensity. We re-ran our global, biome-level, and disturbance models including the proportion of non-native species per plot (the ratio of unique non-native species to the total species richness) as a covariate. While we acknowledge that plant cover or basal area would provide a more appropriate metric, this data does not exist for certain functional groups of plants in our dataset. Ultimately, the inclusion of invasion intensity did not alter our primary conclusions. Our results remained consistent across almost all biomes, with the exception of Xeric deserts, where some shifts in significance were observed (NM vs. mycorrhizal: from more NM shifted to be non statistically significant and NM vs. AM: from non-significant shifted to be more AM). We do not report these model results in the text as the metric of invasion intensity is suboptimal, but would be happy to do so upon the editor's request.

Finally, we agree that the specific distribution of mycorrhizal types in the recipient community is a vital area for future research. While a full mechanistic exploration of "mycorrhizal matching" (i.e. whether native mycorrhizal type mediates non-native mycorrhizal types) is beyond the scope of this global synthesis, we have expanded our results and discussion section to better highlight how the native mycorrhizal type may influence which non-native strategies become successful.

> *Results and Discussion [L394]: This highlights how NM success may be contingent upon the native community's mycorrhizal type and the disruption of local fungal-mediated feedbacks.*

I suppose these comments indicate that I as a reader see clear merit in the MS, without doubt, and understand that in such a global analysis it is difficult to address many (more detailed) research questions and explorations. Hence, it can serve as an important step forward towards answering these more ultimate questions, and provide impetus in showing there is no general answer to the question whether invaders tend to be one mycorrhizal type over the other.

We thank the reviewer for seeing value in this global scale assessment of the role of mycorrhizal type and disturbance in mediating plant invasions. We further agree that this work represents an important starting point for future types of questions and approaches to advance the plant-mycorrhizal invasion field.

Other comments:
L119: the number of plots reported here contrasts with the number in L136; I think the reason for this disparity maybe versions of the database, or inclusion criteria, but whatever the reason would be good indicate more clearly to avoid confusion.

The disparity in plot numbers arises from the distinction between the total scope of the sPlot database and our final analytical subset. As noted in L136, the full sPlot database (v.4.0) contains approximately 2.5 million vegetation plots. However, for the purpose of this study, we applied several rigorous inclusion criteria—such as the requirement for plot-level coordinates, native/non-native status of all recorded species, sufficient mycorrhizal type data across plot floras, and intersection with our environmental rasters—to ensure data quality. This filtering process resulted in the final dataset of 440,778 plots reported in L119. For full transparency, we have added a description of unique plot locations and species remaining after several major data cleaning/processing steps:

> *L144: We used vegetation-plot data from the sPlot database (version 4.0; see Bruelheide et al. 2019; Damasceno et al. in preparation), a global compilation of 2.5 million georeferenced plot surveys conducted from 1888 to 2022 across all major biomes of the world (plot size from 1 to 1,000 m2)*
>
> *L156: To minimize uncertainty in native status classification, we excluded plots in which any occurring species had conflicting native status assignments between the two databases. For plots resampled several times, we only kept the most recent census. Based on this filtering, we retained 65,438 plant species across 2,231,767 vegetation plots (from 2,536,019 initial plot observations).*
>
> *L178:. To ensure sufficient mycorrhizal data at each plot included in the analyses, only plots with at least 80% of species assigned to a mycorrhizal status were considered, retaining 62,145 plant species and 1,867,058 vegetation plots*
>
> *L186: Finally, we retained only plots where non-native plant species occur (non-native count > 0). This resulted in a final dataset that included 440,788 vegetation plots with 39,731 plant species from 14 biomes, sampled between 1899 and 2022 (Supplementary Figure S2).*

L325: here it is stated that "most temperate regions are normally dominated by ectomycorrhizal plants" – I think this is an overstatement. One could argue this to be the case for boreal forests perhaps, but temperate regions are very often mixed, especially when not forested.

We agree with the Reviewer. While EcM *trees* are dominant in many temperate forests, many temperate grasslands and non-forested areas are dominated by AM or mixed strategies. Following the reviewer suggestion to rephrase this section (next comment) we now refer to the EM dominance of native species in forested biomes; this reads as follows:

> [L370] "… Mycorrhizal species were overrepresented in non-native relative to native species in temperate moist broadleaf forests, mediterranean forests, temperate coniferous forests, boreal forests, and tundra biomes, which are generally dominated by EM plants.…"

L330-337: this section is fairly repetitive with redundant statements, best proofread to increase readability.

The first paragraph of this section is focused on the global scale mycorrhizal strategy of non-native species and to note how biome level patterns vary from this. The second paragraph (previously L 330- 344) is then focused on understanding where mycorrhizal non-native species are overrepresented, which mycorrhizal types are they predominantly composed of. We have edited these two paragraphs to clearly split between (1) global and biome level broad mycorrhizal strategy of non-native plants and (2) where there is a mycorrhizal strategy, which mycorrhizal type is principally contributing to this pattern.

> *[L360]: At a global scale, we found that there is a higher probability that non-native species are mycorrhizal compared to native species (Figure 2 B&C), suggesting that mycorrhizal associations may confer an advantage for non-native species. However, this pattern varied strongly across biomes. The global pattern was recovered in most forested temperate regions, where non-native species are predicted to be more mycorrhizal than native species. However, in most grasslands and drylands, we found a higher probability that non-native species are non-mycorrhizal compared to native species (Figure 2A,B,C). These biome-level results suggest that mycorrhizal strategy of non-natives is context-dependent.*

> *[L369]: Beyond the broad mycorrhizal strategy of non-native species (i.e. mycorrhizal), we found that the specific mycorrhizal strategy (i.e. type) varied across biomes. Mycorrhizal species were overrepresented in non-native relative to native species in temperate moist broadleaf forests, mediterranean forests, temperate coniferous forests, boreal forests, and tundra biomes, which are generally dominated by EM plants. In these regions, we found that non-native species were more likely to be AM than NM relative to native species. Further, non-native plant species were more likely to be NM than EM relative to native species in these regions, except for temperate broadleaf and mixed forests, where EM were overrepresented. This suggests that, where non-native plants are more likely to be mycorrhizal, they are also more likely to be AM, except for temperate broadleaf and mixed forests, where non-natives are predicted to associate more with both AM and EM compared to natives. The increased probability of non-native species to be mycorrhizal and primarily AM in these biomes aligns with the more ruderal state of mostly AM understory in these systems. That is, the species that are more likely to invade are AM herbs. Where EM strategies are overrepresented compared to NM, these species may promote success in forested biomes where they are ecologically dominant within the native community (Steidinger et al., 2019).*

L405-406: here there is mention of FM species/plants conferring an advantage to non-native establishment. This suggests it is the strategy of the recipient community that provides the advantage, whereas the strategy of the invader is what's meant.

We agree that this was not as clear as it could have been; we have therefore revised the sentence to clarify that the strategy is for non-native species. The reads as follows: L450:

> *"The facultative mycorrhizal (FM) strategy has been identified as a key driver of naturalization success…"*

L420: here it is stated that while FM plants tend to be flexible and have broad ranges, they are in fact underrepresented as invasives. On the one hand, there is an implicit bias towards FM designation in more common plants: species can have 0, 1, or more entries in a database as either being colonized by mycorrhizal fungi or not. Only those with sufficient entries can be designated FM (have entries of both being mycorrhized or not), hence there is an increasing chance to be designated FM with increasing entries, as it only takes one count of the opposite type to become FM instead of myc vs NM (mistake or not). On the other hand, common plants may be more likely to have already been naturalized (and so don't count as invasives, but count in the denominator). These considerations don't necessarily negate the conclusion, but are important to keep in mind when drawing ecological conclusions.

We agree that mycorrhizal assignment can be biased toward facultative mycorrhizal (FM) status when contrasting reports are available for a given species. However, it is important to clarify that FM designation does not necessarily arise from the accumulation of multiple independent records: a single study typically analyses several plant individuals or populations, and documented intra-specific variability (e.g. some individuals colonized and others non-mycorrhizal) can already lead to an FM assignment. Conversely, many widespread and well-studied species with numerous records retain a consistent mycorrhizal or non-mycorrhizal classification. Such biases are inherent to large mycorrhizal trait databases and apply broadly across plant species (Soudzilovskaia et al., 2020). To acknowledge this, we add a sentence in the Limitations and future directions section; this reads as follows:

> [L498]: *Further, these assignments may reflect both genuine intra-specific variability and limitations in sampling intensity or diagnostic resolution (e.g. for FM), which can introduce uncertainty in mycorrhizal classification.*

L538: yellow should here be green?

We have corrected this mistake in the Figure caption.

<u>Reviewer 3</u>

The manuscript entitled "Mycorrhizal strategy of non-native plants varies with biome and disturbance" is based on an impressive global comparison of mycorrhizal strategies between native and non-native floras. The authors also assess the influence of biomes, disturbances and their interaction on native vs. non-native plants mycorrhizal strategies. To address these questions, they use the most up-to-date global databases on vegetation plots (the sPlot database), naturalized non-native plants (Global Naturalized Alien Flora) and native plants distributions (Plants of the World Online), as well as plants mycorrhizal strategies (FungalRoot database). This type of research is fundamental to help us understand the biogeographic patterns of plant invasions. However, I found some issues with the manuscript

We thank the reviewer for the constructive and detailed feedback. We appreciate the recognition of our study as a key step in understanding the biogeographic patterns of plant invasions using the most up-to-date global databases. We have integrated all comments where possible, and believe these changes have improved the clarity and impact of our work.

As I understand (according to lines 190-191) the study is based only on plots where non-native species have become naturalized. In these plots, the presence of non-native plants may have caused a shift in the dominant mycorrhizal strategy of native plants (for example, if some native species have been displaced due to competition with non-native species, and only a particular subset of native species remain in each plot). For instance, in some contexts it is possible that only obligate ectomycorrhizal species are able to compete with non-natives and other mycorrhizal types (such as arbuscular mycorrhizal) have been replaced in invaded plots. As a result, the comparison of mycorrhizal strategies between the non-native flora and the remaining native flora for each plot would not be particularly informative of how advantageous the non-native mycorrhizal strategy was for non-native plant establishment. Therefore, I suggest mycorrhizal strategies for native species should be assessed in plots where non-natives are absent (and also their influence on natives). One option would be to use paired uninvaded plots (100% native) vs invaded plots (non-native > 0).

We agree that this issue is vital to consider. As the reviewer suggests, the establishment of non-native species may shift the resident community's mycorrhizal profile, potentially masking the baseline difference between native and non-native strategies. To address this, we performed a validation analysis comparing the mycorrhizal proportions of native species in invaded plots (non-native count > 0) versus uninvaded (100% native) plots at each biome. While this comparison provides valuable insights, we recognize it may contain intrinsic bias; uninvaded plots often remain so because they occupy distinct niches (e.g., extreme soil types or remote geographic locations) that are not representative of the 'invadable' landscape. Nevertheless, as a starting point in the absence of long-term temporal data, this analysis allows us to apply some level of correction for potential changes in the native mycorrhizal type composition due to invasion. Our results (detailed in the Methods and Limitations section and Supplementary Table S7) show that our primary conclusions remain consistent even after this adjustment, with only minor shifts in one specific biome (Mediterranean woodlands). We believe this validation demonstrates that our findings are robust to the biotic changes caused by invasion itself.

We added a section in the Methods where we explain the rationale and the methodology used to test for potential shifts in the resident community's mycorrhizal profile due to invasion:

[L332] Impact of invasion on the native plant mycorrhizal type composition

> *The displacement of native species by non-natives can drive non-random shifts in functional community structure, potentially skewing baseline mycorrhizal patterns (Vilà et al., 2011; Pyšek et al., 2012). To assess this impact, we modeled the proportions of mycorrhizal types (NM vs Myc) in native species between invaded plots and uninvaded plots using a generalized linear model with a binomial error distribution and a logit link function. The model included an invasion status by biome interaction and environmental variables as covariates. Results showed that the proportion of NM species in uninvaded plots was generally higher than in invaded plots (Supplementary Table S6). To assess whether this shift in native mycorrhizal types due to invasion would qualitatively alter our main results and conclusions, we adjusted our original native NM estimates using a biome-specific correction factor derived from the new model described above. This sensitivity analysis (Supplementary Table S7) confirmed that, although invasion alters local mycorrhizal composition, our primary conclusions remain robust to these shifts.* We also highlight that future work should expand on this line of inquiry, noting that resampling initially uninvaded and subsequently invaded plots may be a path forward.

[L487-491]:

> *However, non-native establishments may alter the native community's mycorrhizal type composition. When we accounted for this effect at the biome level, our results remained largely consistent (Supplementary Table S7). While this assessment aligns with our major conclusions, this highlights the value of plot resampling to compare native communities in uninvaded and subsequently invaded locations.*

Both in the introduction and the discussion sections, the authors fail to describe the main findings of previous relevant literature addressing the same question. In the introduction (lines 73-75) the authors mention that “the role of mycorrhizal strategy in invasion biology remains understudied, and it is unclear which mycorrhizal strategy is associated with non-native plant establishment across different biogeographic contexts”, but previous studies have found consistent results showing that facultative mycorrhizal species show higher naturalization success (i.e. facultative mycorrhizal species are more likely to become naturalized, and have become naturalized in more regions or over a larger area than obligate mycorrhizal species and non-mycorrhizal species) (Menzel et al. 2017, Pyzek et al. 2019, Moyano et al. 2020).

In the discussion, the authors state that “facultative mycorrhizal plants do not confer an advantage to non-native establishment” without considering previous studies showing the opposite (Menzel et al. 2017, Pyzek et al. 2019, Moyano et al. 2020). The authors should discuss how their results contrast with previous studies focused on the same question (addressed by different approaches).

The apparent contradiction between previous findings and ours in relation to FM plant species warrants particular attention. We agree that our findings regarding FM species need to be more carefully contextualized against the seminal works of Menzel et al. (2017), Pyšek et al. (2019), and Moyano et al. (2020). Yet, we would like to highlight that we are using a different metric of mycorrhizal strategy of non-natives species relative to the native ones, within the same plot (and geographical and environmental context).

Previous studies have largely focused on naturalization success at the species level (e.g., whether an FM species is more likely to establish *at least one* population outside its range). This body of work has found that FM plant species have larger ranges than other mycorrhizal types (Menzel et al., 2017; Moyano et al., 2020). These analyses evaluate non-native or invasive plant species and subsequently assess which mycorrhizal type is most prevalent in non-native floras (often the number of study regions into which it has invaded). This tells us that FM plant species tend to occur in larger ranges, and likely are able to occupy wide environmental niches. This is consistent with the flexible nature of FM plant species.

This commonly used approach, however, does not compare non-native strategy to the native strategy – do non-native employ a significantly different strategy than native plant species? Our study examines community-level dominance (the relative abundance/frequency of these types compared to the local native flora) (Cadotte & Lovett-Doust, 2001; Crawley et al., 1996; Pyšek & Richardson, 2007). This comparison between native and non-native floras within the same geographic region can identify which mycorrhizal types are more widespread

in the non-native relative to the native subsets of the flora. Importantly, comparing floras within the same geographic region inherently allows us to control for biogeographical factors (van Kleunen et al. 2010; Pyšek & Richardson 2006).

While FM species may be highly successful at naturalizing globally due to their flexibility (as shown by Menzel et al.), our data suggests they are frequently underrepresented in local plot-level compositions relative to the resident native species in many biomes.

We have expanded on this difference and commentary information gained from this approach in the Introduction and Discussion.

Introduction:

> [L110-119]: *Until now, few studies have directly compared the prevalence of mycorrhizal strategies between non-native and native species within the same communities at large spatial scales (Cadotte & Lovett-Doust, 2001; Crawley et al., 1996; Pyšek & Richardson, 2007; van Kleunen et al., 2010). Instead, studies largely focus on the non-native flora in isolation from native flora, assessing the proportion of non-native species of each mycorrhizal type (Menzel et al., 2017; Moyano et al., 2020). Importantly, this precludes testing whether the mycorrhizal strategy of non-natives differs from the dominant strategy of native plant species (i.e., within a local community sharing biogeographical and environmental context). The few regional studies that do test for differences between the strategy of non-natives versus natives report contrasting results.*

Discussion: We revised the discussion paragraph [L449-474] to more explicitly compare our results to the previous studies by Hempel et al., 2013, Menzel et al., 2017, Moyano et al. 2020 and Pyzek et al. 2019.

> *L449: Facultative mycorrhizal strategy does not confer an advantage to non-native establishment*
>
> *L450: "The facultative mycorrhizal (FM) strategy has been identified as a key driver of naturalization success, with FM species typically establishing in more regions and over larger geographic areas than obligate or non-mycorrhizal species (Menzel et al., 2017; Moyano et al., 2020; Pyšek et al., 2019). This suggests that the flexibility to switch between mycorrhizal states provides the broad environmental niche necessary for global range expansion. However, our plot-level analyses show that FM species are consistently underrepresented among non-native compared to native plants (Figure 4 A&C)."*
>
> *L467: … "Contrary to expectations based on the broad ecological flexibility and wide ranges typically associated with FM species (Hempel et al., 2013; Menzel et al., 22017; Moyano et al. 2020; Pyšek et al., 2019), we found FM species to be underrepresented among non-natives at the plot level. This suggests that the FM strategy does not confer a consistent advantage in invasion. By directly benchmarking non-native strategies against co-occurring natives, we demonstrate that local invasion success is not merely a product of inherent ecological plasticity, but rather depends on how a species' strategy diverges from the established native baseline."*

Line 39-40: This is only true for one metric of disturbance, while the other metric shows the opposite trend. These contrasting results should be clearly described in the abstract.

We agree that a broad generalization that disturbance favors NM types is not warranted given the divergent patterns observed between our two metrics. It is indeed interesting that these two metrics influence mycorrhizal strategy of non-native species in different ways.

These indices reflect different disturbance pressures on plant-fungal associations. SDI (generalized disturbance) reflects the frequency and intensity of episodic events (e.g., fire, grazing, flooding) that likely mechanically disrupt soil fungal structures. This likely degrades the mycorrhizal community and reduces mycorrhizal propagule pressure (Hogberg & Piearce, 1986; Sheldrake et al., 2017), favoring the NM strategy in non-native plants. In contrast, HMI (human disturbance) reflects stable anthropogenic infrastructure and human pressure. In these systems, mycorrhizal fungal communities may persist or be actively spread by human activity (e.g. via overwhelmingly mycorrhizal crops and ornamental species), potentially favoring mycorrhizal non-natives. We tested the correlation between the SDI and HMI and find that while the relationship is statistically significant ($p < 0.001$) due to the high power of our dataset, the correlation is negligible (Pearson's $r = -0.03$). This confirms that these metrics capture fundamentally distinct ecological and anthropogenic dimensions.

Following the Reviewer's suggestion (as well as that of Reviewer 2), we have carefully updated the manuscript to ensure the difference in metrics and associated interpretation of results is reflected throughout:

L 39-42 "Disturbance type and intensity emerged as key drivers of mycorrhizal strategy of non-native species. Higher generalized disturbance predicted non-native species to be more non-mycorrhizal, while higher human disturbance predicted non-native species to be more mycorrhizal.

Section Titles [Line 404]: "Mycorrhizal strategy of non-native plants depends on disturbance intensity and type"

Results
On the effect of SDI [Lines 405-429]: "We found that generalized disturbance (SDI) increases the probability of non-native species being non-mycorrhizal (NM) compared to natives at the global level and in the majority of biomes (Figure 3 A&C). This pattern was especially pronounced in tropical grasslands and shrublands, deserts and xeric shrublands, and tropical moist broadleaf forests. These biomes are typically dominated by arbuscular mycorrhizal (AM) associations (Read, 1991), yet are increasingly subject to natural (e.g., fire, grazing) and anthropogenic (e.g., land conversion, deforestation) disturbances (Morris, 2010; Saygın et al., 2011; van Etten et al., 2022; Whitmore & Burslem, 1996). Since AM fungi have limited dispersal capacity (Kivlin et al., 2011), such disturbances can degrade AM fungal inoculum, reducing establishment opportunities for AM-dependent species. Consequently, NM species – often ruderal, disturbance-adapted, and unconstrained by symbiont dependence – can rapidly exploit these conditions (Brundrett, 1991; Peat & Fitter, 1993). This helps explain the consistent rise of NM strategies among non-natives in highly disturbed AM systems (Reinhart & Callaway, 2006; Richardson et al., 2000; Vogelsang & Bever, 2009). In contrast, the opposite pattern was found in Mediterranean forests: increased disturbance (SDI) was associated with higher proportions of mycorrhizal species among non-natives (Figure 3A&C). Unlike AM-dominated systems, these forests often host ectomycorrhizal (EM) or mixed AM-EM long-lived woody perennials (Gerz et al., 2019; Read, 1991). These systems may thus retain functional mycorrhizal associations even under disturbance due to the resilience of trees and associated EM fungi, which produce resistant propagules, disperse more effectively, and can persist independently of hosts (Horton & Horton, 2017; Policelli et al., 2022; Smith & Read, 2008). Specifically, the frequent fire and drought of Mediterranean ecosystems may have selected for EM spore banks that are more resilient than those in boreal or temperate forests, where disturbance leads to severe fungal biomass decline (Buscardo et al., 2010; Dahlberg, 2002; Livne-Luzon et al., 2021)."

On the effect of HMI [Lines 431-447]: "The Human Modification Index (HMI), reflecting more stable human pressures such as urbanization and infrastructure, was generally associated with increased prevalence of mycorrhizal species among non-natives (Figure 3 B&D; Supplementary Figure S4 A&B). These results align with results by Clavel et al., (2024), who showed that roadside disturbance promoted mycorrhizal species. Specifically, increasing human disturbance was associated with a higher proportion of mycorrhizal non-natives in grasslands, tropical moist broadleaf forests, boreal forests, and tundra, biomes that were more likely to be non-mycorrhizal under high SDI. In contrast, for Mediterranean and temperate forest biomes (both coniferous and broadleaf), increased human disturbance was associated with higher proportions of non-mycorrhizal non-natives. These contrasting patterns between SDI and HMI underscore the need to consider multiple disturbance types when assessing invasion dynamics. Independence from mycorrhizal fungi may confer an advantage in systems with degraded fungal communities or altered nutrient regimes, where NM species can bypass the carbon costs of symbiosis (Hogberg & Piearce, 1986; Sheldrake et al., 2017; Yu et al., 2022). In contrast, associations with mycorrhizal fungi may confer an advantage to non-native species in less frequently disturbed, human-modified systems where fungal partners persist or are actively spread through human activities (e.g. mycorrhizal crops and ornamental species)."

We emphasize that these interpretations reflect broad-scale associations rather than direct causal mechanisms, and are intended to motivate targeted regional, experimental, and trait-based studies that can explicitly test these processes.

Conclusions [Lines 515-521]: "Moreover, our results show that while disturbance is a key predictor of mycorrhizal strategy, the direction of this effect hinges on the nature of the disturbance. Specifically, generalized disturbance increases the proportion of non-mycorrhizal, whereas human disturbance favors mycorrhizal non-native species. These results suggest distinct underlying mechanisms: while

sustained and frequent disturbance likely depletes fungal inoculum, stable anthropogenic pressures appear to preserve, or even facilitate, mutualisms between fungi and non-native plant species."

Line 83-85: I suggest the authors also cite Nuñez et al. (2009) here

We have added this citation here [L90]

Lines 85-89: Menzel et al. (2017) and Moyano et al. (2020) found evidence that facultative mycorrhizal species show higher establishment success outside their native range (i.e. have become established in more regions or a larger area) than obligate mycorrhizal and non-mycorrhizal species. As it is written this sentence ignores their main findings.

We have revised the Introduction [Lines 89-91] to explicitly acknowledge the findings of these studies: that facultative mycorrhizal (FM) species have been shown to establish across a larger number of regions (i.e. show a larger invasion range).

> *[L92]: Consequently, NM and FM strategies may be more optimal for non-native plant species. Indeed, regional and global-scale studies have demonstrated that FM non-native species have the highest naturalization success in mainland regions (Menzel et al., 2017; Moyano et al., 2020).*

We also expand both the Introduction and Discussion sections to highlight how the approach of our study differs and provides additional information from this previous approach. Please see prior response to comment regarding this for more details and associated text.

Line 135: Is there a standardized plot size for the sPlot database? If not, what is the range of plot sizes included in this database?

The sPlot database integrates data from vegetation surveys of contiguous areas with varying plot sizes, ranging from 1-1,000 m2. We added this info in the Methods section [L146-147]:

> *We used vegetation-plot data from the sPlot database (version 4.0; see Bruelheide et al. 2019; Damasceno et al. in preparation), a global compilation of nearly 2.5 million georeferenced plot surveys conducted from 1888 to 2022 across all major biomes of the world (plot size from 1 to 1,000 m2).*

Lines 154-156: Why not consider mycorrhizal types orchid mycorrhizal and ericoid mycorrhizal?

These mycorrhizal types were excluded from the analysis due to their high host-specificity, geographic restriction, and marginal representation within the sPlot database. Specifically, based on assignments from the FungalRoot database—which itself contains only 1% orchid and 2% ericoid associations—these types accounted for just 0.02% and 0% of the species in our dataset, respectively. The resulting zero-inflation and lack of statistical power precluded robust modeling of these strategies, and their inclusion would not have yielded broadly applicable insights for most plant taxa.

> We added this explanation in the method section:
>
> L175 "*Although ecologically relevant, orchid (ORC) and ericoid (ErMF) mycorrhizal types were excluded from our analyses because they represent a very small percentage of the global flora in the sPlot database (sPlot species assigned: ORC = 0.02%) or were absent at all (ErMF = 0%).*"

Line 379: This sentence contradicts the first sentence of the previous paragraph (lines 362-364), where the authors state "…a trend that was consistent across global, and biome level analyses".

We appreciate the Reviewer for pointing out this issue. We agree that this phrasing obscures the nuances between different biomes and the distinct patterns captured by the two disturbance metrics (SDI vs. HMI).

To resolve this, we have revised the text in both sections to be more precise:

> [L405] : *We found that generalized disturbance (SDI) increases the probability of non-native species being non-mycorrhizal (NM) compared to natives at the global level and in the majority of biomes (Figure 3 A&C).*

Lines 382-387: This is an interesting point, but it should apply not only to mediterranean forests but also to temperate forests and boreal forests.

Pasting here previous text: "Forest biomes often host ectomycorrhizal (EM) or mixed AMEM species, many of which are long-lived woody perennials (Gerz et al., 2019; Read, 1991). These systems may thus retain functional mycorrhizal associations even under disturbance due to the resilience of trees and associated EM fungi, which produce resistant propagules, disperse more effectively, and can persist independently of hosts (Horton & Horton, 2017; Policelli et al., 2022; Smith & Read, 2008)."

We agree that this could apply to forested biomes in general; we have revised the Discussion to note this [Lines 418-426]:

> *In contrast, the opposite pattern was found in Mediterranean forests: increased disturbance (SDI) was associated with higher proportions of mycorrhizal species among non-natives (Figure 3A&C). Unlike AM-dominated systems, these forests often host ectomycorrhizal (EM) or mixed AM-EM long-lived woody perennials (Gerz et al., 2019; Read, 1991). These systems may thus retain functional mycorrhizal associations even under disturbance due to the resilience of trees and associated EM fungi, which produce resistant propagules, disperse more effectively, and can persist independently of hosts (Horton & Horton, 2017; Policelli et al., 2022; Smith & Read, 2008).*

Furthermore, we have added a clarifying sentence [Line 426] noting that Mediterranean ecosystems may possess more fire-resistant EM spore banks than boreal or temperate systems, consistent with studies showing their unique evolutionary resilience to frequent disturbance (Buscardo et al., 2012; Livne-Luzon et al., 2021).

> *Specifically, the frequent fire and drought of Mediterranean ecosystems may have selected for EM spore banks that are more resilient than those in boreal or temperate forests, where disturbance leads to severe fungal biomass decline (Buscardo et al., 2010; Dahlberg, 2002; Livne-Luzon et al., 2021).*

Lines 389-390: Again, this sentence contradicts the first sentence of the first paragraph in this subsection (lines 362-364), where the authors describe the consistent effect of disturbance on mycorrhizal strategy

We revised the first sentence in the first paragraph and replaced the term 'consistent' with a more precise description of the trends to remove the potential contradiction. Please refer to the answer to the previous comment.

Line 405: The title of this subsection is too strong and definitive. While the authors found that facultative mycorrhizal species are underrepresented when comparing non-native vs native species, other approaches have found evidence that facultative mycorrhizal species show higher naturalization success (i.e. are more likely to become naturalized, have become naturalized in more regions or over a larger area than obligate mycorrhizal species and non-mycorrhizal species) (Menzel et al. 2017, Pysek et al. 2019, Moyano et al. 2020). The authors should discuss how their results contrast with previous relevant literature on the same question (addressed by different approaches).

We agree that explicitly engaging with these previous studies and findings is important. Previous work used a different approach to our mycorrhizal invasion strategy assessment here (non-native relative to native mycorrhizal types). Please see detailed response and associated changes to the text to address this concern above. Reporting lines of interest in the results and discussion section: 450-454, 455-456 and 467-474

Lines 454-455: This is only true for one metric of disturbance, while the other shows the opposite trend. These contrasting results should be clearly stated in the conclusion.

We agree that the contrasting effects of SDI and HMI are a fundamental finding of this study. Please refer to our detailed response above where we provide an explanation for these divergent patterns and discuss the negligible correlation between the two metrics ($r = -0.03$), which confirms they capture distinct ecological dimensions. To ensure this is clearly communicated to the reader, we have updated both the Abstract and Conclusion sections to avoid broad generalizations and instead highlight the metric-dependent nature of the results:

Abstract

[L39-42]: *Disturbance type and intensity emerged as key drivers of mycorrhizal strategy of non-native species. Higher generalized disturbance predicted non-native species to be more non-mycorrhizal, while higher human disturbance predicted non-native species to be more mycorrhizal.*

Conclusion

[L515-521] : *Moreover, our results show that while disturbance is a key predictor of mycorrhizal strategy, the direction of this effect hinges on the nature of the disturbance. Specifically, generalized disturbance increases the proportion of non-mycorrhizal, whereas human disturbance favors mycorrhizal non-native species. These results suggest distinct underlying mechanisms: while sustained and frequent disturbance likely depletes fungal inoculum, stable anthropogenic pressures appear to preserve, or even facilitate, mutualisms between fungi and non-native plant species.*

Lines 456-460: It is not only an assumption or an idea, there is evidence from previous literature showing that facultative mycorrhizal species are more likely to become naturalized outside their native range and across a wider area (Menzel et al. 2017, Pyzek et al. 2019, Moyano et al. 2020). Further, while the authors provide evidence that facultative mycorrhizal species are underrepresented when comparing non-native vs. native flora, this does not necessarily refute previous findings addressing this same question using different approaches.

We agree that making this distinction of previous tests and findings compared to those in the present study is important. We have made several edits throughout the text to clarify differences and inferences that can be drawn from the distinct approaches. Please see response to previous comment addressing this concern.

We would like to express our sincere gratitude to the Reviewers and the Editor for their constructive feedback and support throughout the review process. We are pleased that the Reviewers are mostly satisfied with our revisions and have found the manuscript to be a valuable contribution to the field. Following the final suggestions from Reviewer 1 and the Editor, we have further refined the manuscript to ensure that the framing of our study accurately reflects its observational and correlative nature, while maintaining the rigor of our biogeographic investigation.

Reviewer #1

Regarding the novelty of this study, the causative inference of this study is necessarily limited. This is inherent to all such studies using plot level data like this. The introduction still claims to be testing hypotheses. I believe this is misleading, given the inherent correlative nature of this study. I applaud the authors for their discussion of these limitations in the Discussion section, but the Introduction should be tempered to better reflect the actual study.

We agree with the Reviewer (and the Editor) that distinguishing between observational associations and experimental causal testing is crucial for the framing of this work. We have tempered the language in the final paragraph of the Introduction to acknowledge the inherent limitations of plot-level observational data. We now frame our work as an investigation of biogeographic patterns that provides a robust foundation for future mechanistic research.

L126: *We revised the text replacing 'test' with "investigate", which is less associated with experimental designs.*

L130: *We revised the text to: "we use statistical analyses..." to emphasize the observational methodology.*

L137: *We revised the final paragraph to state: "While our observational approach identifies large-scale biogeographic patterns rather than direct causal mechanisms, these statistical associations provide compelling evidence for how mycorrhizal strategies relate to non-native plant establishment. Furthermore, they generate robust, testable hypotheses for future experimental studies to mechanistically disentangle how these fungal symbioses underpin the invasion process across global environments."*

L315 (Discussion): *We added a reinforcing statement in agreement with the Introduction: "Finally, our study is correlational in nature, and experiments will be needed to mechanistically test for invasibility of different mycorrhizal types under different biotic and environmental conditions."*

I appreciate the inclusion of a sentence on mycorrhizal colonization in the limitations section, but more in depth description as to how to differences in mycorrhizal reliance could influence these patterns would be helpful.

We thank the reviewer for this suggestion. We have expanded the "Limitations" section to provide a more nuanced discussion on mycorrhizal reliance and growth response. We explicitly address how context-dependency and varying degrees of dependency may influence establishment barriers.

L308: *“Beyond taxonomic coverage, an ecological limitation of our analysis is that these assignments do not account for quantitative differences in mycorrhizal reliance and growth response. Because the fitness benefits of mycorrhizal associations are highly context-dependent, species with high reliance may face different establishment barriers compared to those with low or facultative reliance, particularly under the varying disturbance regimes and environmental conditions.”*
